# Food-derived compounds targeting ferroptosis for cancer therapy: from effects to mechanisms

**DOI:** 10.3389/fonc.2025.1568391

**Published:** 2025-06-09

**Authors:** Jin-Wei Zhao, Wei-Yi Zhao, Zhongyang Yu

**Affiliations:** ^1^ Department of Hepatopancreatobiliary Surgery of Second Hospital of Jilin University, Jilin University, Changchun, China; ^2^ Department of Cardiology, Jilin Airport Hospital, Jilin, China; ^3^ State Key Laboratory of Cardiovascular Diseases, Shanghai East Hospital, School of Medicine, Tongji University, Shanghai, China

**Keywords:** food-derived bioactive ingredients, ferroptosis inducer, synergistic mechanism, cancer, treatment

## Abstract

Ferroptosis is distinctive type programmed cell death. Tumor cells, with their higher iron levels, render them more susceptible to ferroptosis Inducing ferroptosis can activate immune cells, regulate immune evasion, and inhibit the biology activity of cancer cells. Therefore, ferroptosis-induced cancer cell death could become a promising approach for cancer treatment. Dietary compounds are an important source for drug discovery, and there has been an increasing amount of literature on food-derived ferroptosis inducers and their applications in cancer treatment. This review provides an overview of the regulatory mechanisms involved in ferroptosis, explores the mechanisms by which dietary compounds act as ferroptosis inducers, and discusses their effects on various cancers, especially by accumulating lipid ROS and overloading Fe2+, along with inhibiting GPX4 expression to promote ferroptosis in tumors. Additionally, the latest advancements in new methods for inducing ferroptosis, including the use of nanomaterials, are also summarized. Finally, the challenges and opportunities of developing dietary compounds as ferroptosis inducers are discussed, focusing on the discovery of new targets, enhancing selectivity, as well as reducing toxicity and the recurrence of side effects. As far as we know, this is the first comprehensive and systematic summary on the anticancer effects and mechanisms of food-derived ferroptosis inducers.

## 1 Introduction

According to the most recent estimates from the International Agency for Research on Cancer (IARC), around 20 million new cancer cases were diagnosed worldwide in 2022, including non-melanoma skin cancer (NMSC), and 9.7 million people died from cancer. These estimates suggest that approximately 1 in 5 men or women will eventually be diagnosed with cancer, and about 1 in 9 men and 1 in 12 women will die from cancer (1). Notably, there are notable variations in the age-standardized 5-year survival rates across different cancers. The prognosis is best for thyroid cancer, while pancreatic cancer has the worst prognosis, with a five-year age-standardized survival rate as low as 7.2% (2). Important causes and potentially modifiable risk factors for cancers include infectious agents (viruses, bacteria, and parasites), alcohol, tobacco, diet, physical activity, overweight and obesity, among others (3). The global incidence of cancer has been increasing annually. In 2020, there were more than 19 million reported cases of cancer worldwide, leading to approximately 10 million deaths from the disease (4); in 2012, 14.1 million new cancer cases were reported and 8.2 million deaths from cancer (5). Breast cancer, lung cancer, and colorectal cancer (CRC) are the fastest growing types of cancers globally. The predicted patterns of cancer incidence and mortality can be partially attributed to overall population growth, increased life expectancy, and related risk factors such as changes in diet and smoking habits (6–8). These data highlight the increasing global cancer burden. However, current cancer treatments are limited, as tumor cell resistance diminishes the effectiveness of traditional therapies such as radiotherapy and chemotherapy, posing significant challenges for cancer treatment (9).

Strategies for cancer treatment always prioritize selectively eliminating cancer cells while minimizing damage to healthy cells. Regulated cell death (RCD) is an crucial approach to eliminating cancer cells, and RCD strategies can specifically target cancer cells, boosting the effectiveness of cell death induced by drug while minimizing side effects on normal cells (8). Ferroptosis is a recently identified mode of reactive oxygen species(ROS)-mediated RCD involving the disturbance of cellular redox balance, metabolism of iron and lipid peroxidation (LPO) as core mediation medium. Ferroptosis is defined by the iron-dependent buildup of membrane-localized lipid peroxides, which leads to cell death. It is closely linked to a range of both physiological and pathological processes, Including in human and animal cancers (10, 11). The regulation of ferroptosis could be an effective approach for treating various types of cancer (12–14). Ferroptosis has been revealed as an inherent mechanisms for suppressing tumors (15–17).

Inducing ferroptosis could be a promising approach to eliminate cancer cells and overcome drug resistance in conventional cancer therapies (18). Additionally, ferroptosis could become a therapeutic target for alleviating pathological damage to organ tissue (19). Based on in-depth research on ferroptosis, many clinical medications and chemical substances have been demonstrated to regulate ferroptosis. Drugs that have been shown to induce ferroptosis include chemotherapy drugs such as sulfasalazine and cisplatin, targeted drugs like sorafenib and lapatinib, as well as antibiotics (20–23). However, many of these drugs can lead to endocrine dysfunction, peripheral neuropathy, liver fibrosis, gastrointestinal bleeding, and renal abnormalities, including kidney failure (24).

Food-derived bioactive compounds, which are abundant in fruits, vegetables, grains, seeds, and spices, have been important sources of drug research and development for decades. Accumulating evidence shows that many foodborne compounds and their derivative metabolites have favorable effects on regulating ferroptosis (18). In comparison to certain traditional ferroptosis regulators, food-derived compounds offer the benefits of structural stability, targeting multiple regulatory sites, and low toxicity, which makes the development of natural ferroptosis regulators highly promising (25).

In recent years, substantial advancements have been made regarding natural products that can induce ferroptosis, and natural products have garnered widespread attention in cancer treatment. The aim of the review is to provide the latest information on food-derived compounds that induce ferroptosis in cancer treatment, their molecular targets, and their mechanisms. These compounds are classified on the basis of their characteristic skeletons and mainly include saponins, alkaloids, phenols, and flavonoids. Additionally, this review covers the classical molecular mechanisms and regulatory signaling pathways involved in ferroptosis. Furthermore, this review summarizes the promising prospects of the delivery mediated by nanocarrier of food-derived compounds against cancer and combination therapy involving ferroptosis and other RCD mechanisms, providing new approaches for cancer treatment. Overall, this review not only provides a list of food-derived compounds that can induce ferroptosis to suppress tumors but also offers new perspectives for medicinal chemists and oncologists, helping them discover and develop food-derived compounds that target ferroptosis and/or other RCD mechanisms against cancer.

## 2 Molecular mechanisms of ferroptosis

**Figure 1:**
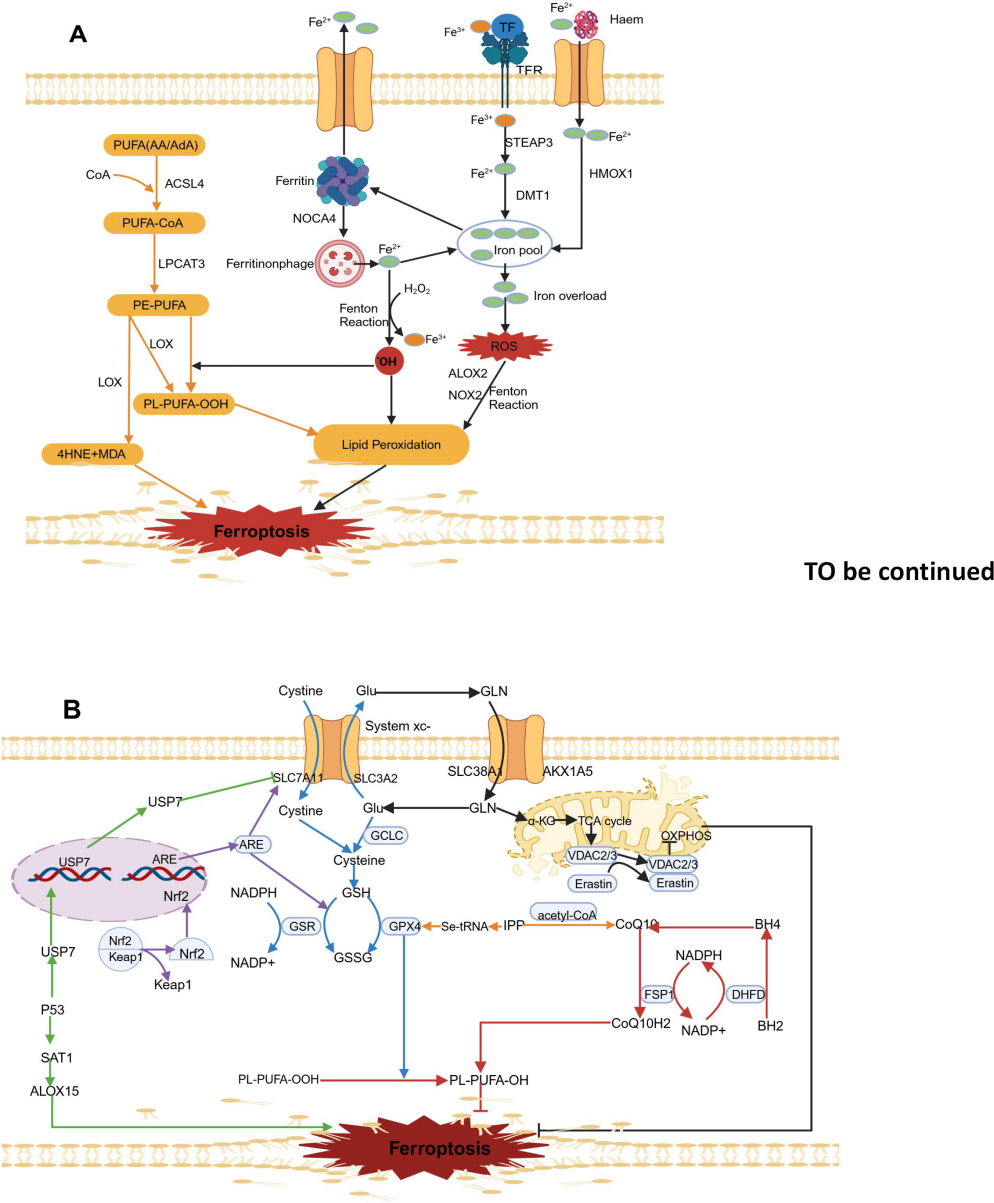
Molecular mechanisms of ferroptosis. **(A)**. Lipid peroxidation (yellow arrow) and iron metabolism (black arrow). **(B)**. Ferroptosis defence mechanisms. The principal ferroptosis related antioxidant defence mechanisms include the Xc^–^-GSH-GPX4 axis (blue arrow), the FSP1- CoQ10-NAD(P)H axis (red arrow), the Nrf2/ARE-GPX4 pathway (purple arrow), the P53 signalling pathway (green arrow), the mevalonate pathway (yellow arrow) and mitochondrial voltage-dependent anion channels (black arrow). The map was created using biorender.

Iron metabolism and LPO (As illustrated in **Figure 1A**) as well as ferroptosis defense mechanisms (as shown in **Figure 1B**) are the three main molecular mechanisms of ferroptosis. The main antioxidant defense mechanisms related to ferroptosis are the xc^–^GSH-GPX4 axis, the FSP1-CoQ10-NAD(P)H axis, the Nrf2/ARE-GPX4 pathway, the P53 signaling pathway, the mevalonate pathway, and mitochondrial voltage-dependent anion channels.

### 2.1 Iron metabolism

As shown in **Figure 1A**, The two oxidation states of iron, Fe^2+^ and Fe^3+^, give iron redox activity. In an oxygen-rich environment, iron can generate ROSthrough the Fenton reaction with various types of phospholipid peroxides and lipid (fatty acid) peroxides (LPO) (26). Typically, circulating Fe^3+^ in the blood is absorbed mainly through the mediation of transferrin receptor 1 (TFR1) and binding with transferrin. Iron bound to transferrin enters cells through endocytosis mediated by receptor. After being taken up, Fe3+ is reduced to Fe2+ in endosomes by the six-transmembrane epithelial antigen of prostate 3 (STEAP3), and then it is transported into the cytosol and free mitochondrial iron pools by solute carrier family 11 member 2(SLC11A2) (27). Various biological processes, including energy metabolism, the synthesis of iron-sulfur proteins in mitochondria, and other metabolic and biochemical activities, require physiological levels of Fe2+ involvement (28). However, excess free Fe^2+^ can cause excessive lipid ROS accumulation through the Fenton reaction and can increase to activate nonheme iron-based arachidonate lipoxygenases (ALOXs) and nicotinamide adenine dinucleotide phosphate oxidases (NOXs), thereby promoting destructive lipid peroxidation (29). To regulate iron balance, excess Fe2+ can be stored in ferritin, which consists of the ferritin light chain (FTL) and ferritin heavy chain 1 (FTH1) (28).Lysosomal degradation of ferritin leads to the release of intracellular Fe2+, which can increase the pool of free iron. Nuclear receptor coactivator 4 (NCOA4)-mediated ferritin degradation is a novel selective autophagy process that releases free Fe2+ and increases susceptibility to ferroptosis (30). Another source of intracellular Fe^2+^ is the degradation of hemoglobin mediated by haem oxygenase 1 (HMOX1). HMOX1 overactivation leads to iron overload in mitochondria and leads to ferroptosis by releasing ferrous ions from haem (31). Additionally, excess intracellular Fe^2+^ can be exported from cells via ferroportin (FPN), which helps maintain iron homeostasis (32). Recent studies suggest transferrin in the liver helps prevent liver damage, fibrosis, and cirrhosis by controlling ferroptosis (33). Additionally, iron regulation pathways influenced by sterol regulatory element-binding protein 2 (SREBP2) contribute to cancer progression, drug resistance, and metastasis, highlighting the importance of iron balance for organ survival (34).

### 2.2 Lipid metabolism

As shown in **Figure 1B**, the key features of ferroptosis include various high levels of lipid peroxides, such as major lipid peroxides (LOOHs), 4-hydroxy-2-nonenal (4-HNE) and malondialdehyde (MDA) (35). The accumulation of lipid peroxides in cell membranes alters membrane permeability and stability, leading to membrane rupture and ultimately triggering ferroptosis (19). Polyunsaturated fatty acids (PUFAs) containing two or more double bonds play a role in the lipid oxidation process during ferroptosis. Through the action of long-chain acyl-CoA synthetase 4 (ACSL4), arachidonic acid (AA) or adrenic acid (AdA) is acylated to form AA/AdA-CoA, which is then converted into phosphatidylethanolamine-arachidonic acid (PE-AA) and phosphatidylethanolamine-adrenic acid (PE-AdA) under the action of lysophosphatidylcholine acyltransferase 3 (LPCAT3). PE-AA and PE-AdA participates in both nonenzymatic and enzymatic oxidative reactions of downstream ferroptosis (36, 37).

The nonenzymatic Fenton reaction refers to the reaction between hydrogen peroxide (H_2_O_2_) and ferrous ions (Fe^2+^)., which generates free radicals such as ferric iron (Fe^3+^) and hydroxyl radicals (OH•). In contrast, Fe^3+^ reacts with superoxide radicals (O_2_•–) and is reduced to ferrous iron (Fe^2+^). These superoxide radicals (O_2_•–) come from NADPH oxidase (NOX) and the mitochondrial electron transport chain (ETC).Mechanistically, nonenzymatic lipid peroxidation starts when OH^•^ derived from the Fenton reaction extracts hydrogen atoms from the double-bonded carbon atoms of PUFAs, forming phospholipid radicals (PL^•^) with a carbon-centered structure. Next, PL• reacts with an oxygen molecule (O_2_), forming phospholipid peroxyl radicals (PLOO^•^). Being a highly reactive radical, PLOO^•^ pulls a hydrogen atom from another PUFA, creating phospholipid hydroperoxides (PLOOH) and generating a new PL^•^. This new radical can then trigger another lipid radical chain reaction (38).

Lipid peroxidation reactions are catalyzed by two types of enzymes: one is lipoxygenase (LOX), which catalyzes the production of toxic products such as MDA and 4-HNE from phosphatidylethanolamine (PE)-PUFAs. These products can react with DNA bases, proteins, and other nucleophilic molecules (39); the other is cytochrome P450 reductase (POR), which promotes lipid peroxidation, leading to ferroptosis (40). Therefore, ACSL4, LOX, and LPCAT3 are important targets for ferroptosis inducers.

### 2.3 Ferroptosis defense mechanisms

#### 2.3.1 xc–GSH-GPX4 axis

Glutathione Peroxidase 4 (GPX4), which is found in the cytoplasm and mitochondria of cells, plays a keyrole in ferroptosis (41, 42). GPX4 is a lipid peroxide enzyme that is dependent on reduced glutathione (GSH) and selenium. In cells, GPX4 uses GHS as a substrate to specifically lower toxic PLOOH to nontoxic phosphatidyl alcohol (PLOH) (43). However, the synthesis of GHS is strictly regulated by the cysteine/glutamate antiporter (system xc**^-^**) located on the cell membrane. System xc**^-^** consists of SLC3A2 and SLC7A11 (xCT), which exchange extracellular cysteine for intracellular glutamate. The inhibition of system xc**^-^** significantly reduces GSH production, thereby decreasing GPX4 activity, leading to lipid ROS build-up and ultimately leading to ferroptosis (44). As a known ferroptosis inducer, RSL3 disrupts the PLOOH neutralization activity of GPX4 by covalently modifying Sec46 (45). The ferroptosis inducer Erastin can inhibit the activity of system xc- to induce ferroptosis (46). In summary, The GSH-GPX4 axis is regarded as the most important negative regulatory factor in ferroptosis (**Figure 1B**).

#### 2.3.2 FSP1-CoQ10-NAD(P)H axis

Ferroptosis suppressor protein 1 (FSP1) can inhibit ferroptosis caused by GPX4 deficiency (47). SP1 is a potent lipophilic antioxidant that can capture oxygen free radicals in phospholipids and lipoproteins, CoQ10 is converted into CoQ10H2 through catalysis by SP1 (48). Nicotinamide adenine dinucleotide phosphate (NAD(P)H), a metabolic intermediate, can capture lipid peroxide radicals, suppress intracellular LPO reactions and ferroptosis (48). Additionally, NADPH regenerates nonmitochondrial coenzyme Q10 (CoQH2) through the catalytic action of FSP1, which helps capture LPO and inhibits Ferroptosis that is independent of GPX4 (47). Moreover, NADPH is extremely necessary for the enzyme dihydrofolate reductase (DHFR), dihydrofolate is reduced to tetrahydrofolate (BH4) by DHFR, which not only neutralizes lipid peroxides (LPOs) but also promotes the synthesis of coenzyme Q10 (49). The FSP1-CoQ10-NAD(P)H pathway can effectively inhibit ferroptosis and might work synergistically with the GPX4-GSH axis (**Figure 1B**).

#### 2.3.3 Nrf2/ARE-GPX4 pathway

Current research suggests that Kelch-like ECH-associated protein 1 (Keap1) inhibits the activity of Nuclear Factor Erythroid 2-Related Factor 2 (Nrf2), which is a key regulator of oxidative stress. Under normal conditions, Keap1 and Nrf2 are bound together. In the presence of oxidative stress, Nrf2 detaches from the Keap1 binding site, quickly translocates to the nucleus, and binds to Antioxidant Response Elements (AREs), thereby increasing the expression of downstream target genes and promoting iron metabolism, NADPH regeneration, and GSH metabolism (50, 51). The activated NRF2 increases the SLC7A11 expression, which plays a key role in cellular glutamate exchange and cysteine production (52). Additionally, As catalysts in glutathione synthesis, GCL and GSS are important target genes of NRF2. The overexpression of NRF2 leads to increased intracellular GSH levels and promotes GPX4 expression (53). Nrf2 also interacts with haem oxygenase-1 (HO-1) to inhibit lipid peroxidation (54). Moreover, Nrf2 activation decreases the amount of iron taken up by cells, boosts iron storage, and reduces the production of ROS (55) (**Figure 1B**).

#### 2.3.4 P53 signaling pathway

Recently, P53 was discovered to promote cellular ferroptosis by regulating the expression of ferroptosis- related genes Earlier research has indicated that P53 interacts with P53 response elements in the SLC7A11 gene promoter region, suppressing SLC7A11 expression and enhancing the vulnerability of cancer cells to ferroptosis (15). Further research showed that P53 lowers the monoubiquitination level of histone H2B by encouraging the nuclear movement of ubiquitin-specific protease 7 (USP7), which in turn epigenetically represses the expression of SLC7A11 (56). Recent findings have shown that P53 affects ferroptosis by regulating spermidine/spermine N1-acetyltransferase 1 (SAT1) (57). SAT1 overexpression rapidly depletes putrescine and spermidine, leading to mitochondria-mediated apoptosis and a slowdown in cell growth (58). P53 can promoteSAT1mRNA expression, thereby improving ferroptosis triggered by arachidonate 15-lipoxygenase (ALOX15); this result indicates that ALOX15 is a metabolic target of P53 in ferroptosis (59) (**Figure 1B**).

#### 2.3.5 Mevalonate pathway

GPX4 is a selenium-containing protein with selenocysteine at its active site. The inclusion of selenocysteine into GPX4 depends on a specific transport protein called selenocysteine tRNA (60). However, isopentenyl pyrophosphate (IPP), produced via the mevalonate (MVA) pathway, is crucia for the process of preparing selenocysteine tRNA and creating GPX4 (61). Additionally, IPP, as a precursor to coenzyme Q10, can produce coenzyme Q10 when acetyl-CoA is present (62). Therefore, the system xc–GSH-GPX4 axis and the NADPH-FSP1-CoQ10 pathway are connected through the MVA pathway (**Figure 1B**).

#### 2.3.6 Mitochondrial voltage-dependent anion channels

Glutamate is a crucial raw material needed for GHS synthesis and is primarily taken up through SLC38A1 and SLC1A5 (63). Glutamate–cysteine ligase catalytic subunit (GCLC) initiates glutathione synthesis by linking cysteine and glutamate (64).Mitochondrial metabolism is the primary source of cellular reactive oxygen species (ROS), and the breakdown of glutamine is thought to influence ferroptosis by providing α-ketoglutarate (α-KG) in the mitochondrial TCA cycle (65). By inhibiting the ETC, lipid peroxide accumulation and ferroptosis are reduced, indicating that ferroptosis is associated with abnormal ROS production (17)., or inhibiting the mitochondrial TCA cycle, knocking out voltage-dependent anion channel (VDAC)2/3, which is used for transporting ions and metabolites in eukaryotic cells (66–70) (**Figure 1B**).

## 3 The role and mechanisms of dietary bioactive compounds as ferroptosis inducers against tumors

**Figure 2:**
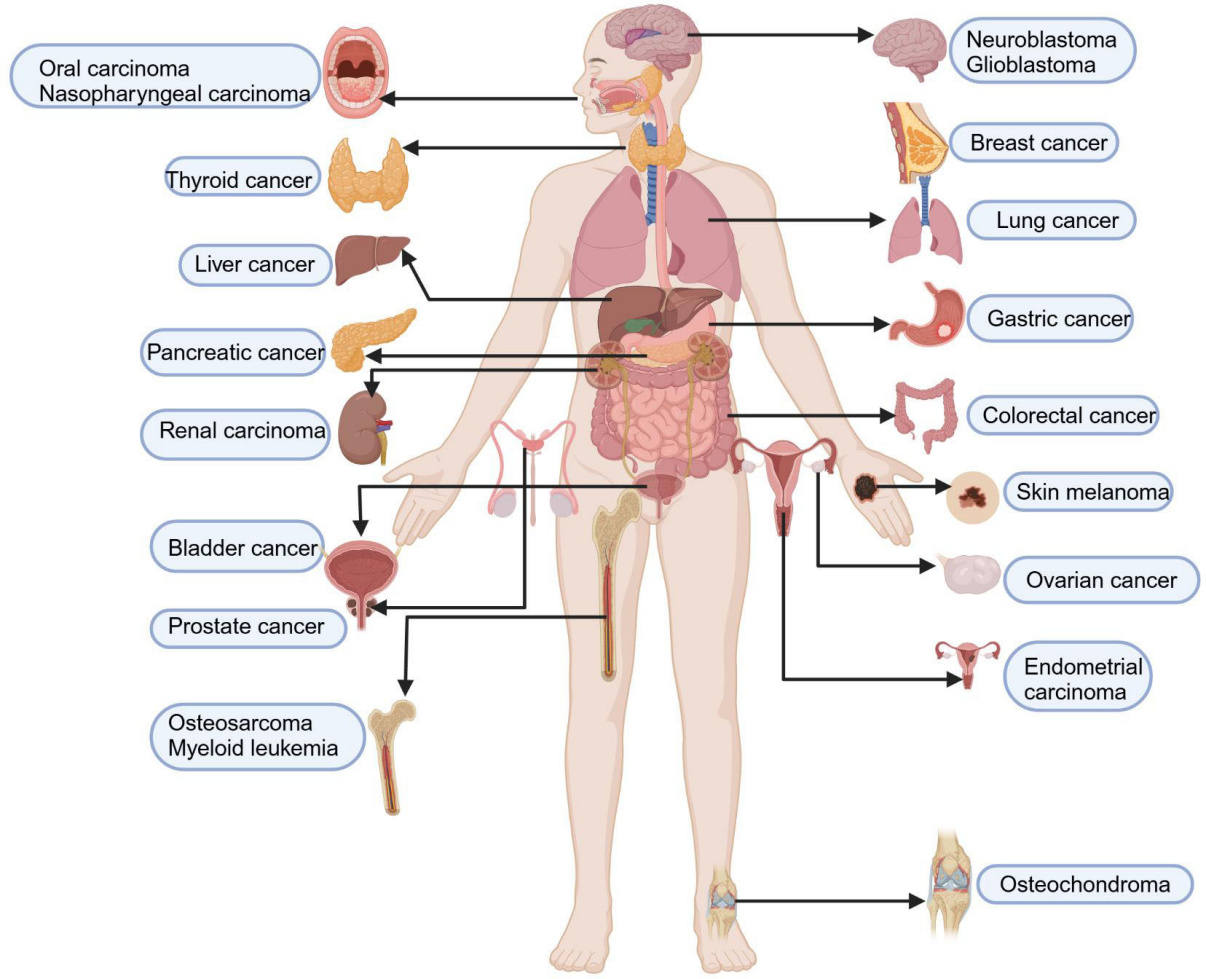
Inhibition of multisystem tumours by food-derived compounds as ferroptosis inducers. Food-derived compounds serve as inducers of ferroptosis, inhibiting the occurrence and progression of multisystem diseases, including tumours of the nervous system (such as gliomas and neuroblastomas), respiratory system tumours (such as nasopharyngeal carcinoma, lung adenocarcinoma, and non-small cell lung cancer), endocrine system tumours (such as follicular thyroid carcinoma), digestive system tumours (including oral cancer, gastric cancer, colorectal cancer, liver cancer, and pancreatic cancer), urinary system tumours (such as kidney cancer and bladder cancer), female reproductive system tumours (such as breast cancer, endometrial cancer, and ovarian cancer), male reproductive system tumours (such as prostate cancer), haematological tumours (such as leukaemia), skin system tumours (such as melanoma), and musculoskeletal tumours (such as osteochondromas and chondrosarcomas). The map was created using biorender.

**Figure 3:**
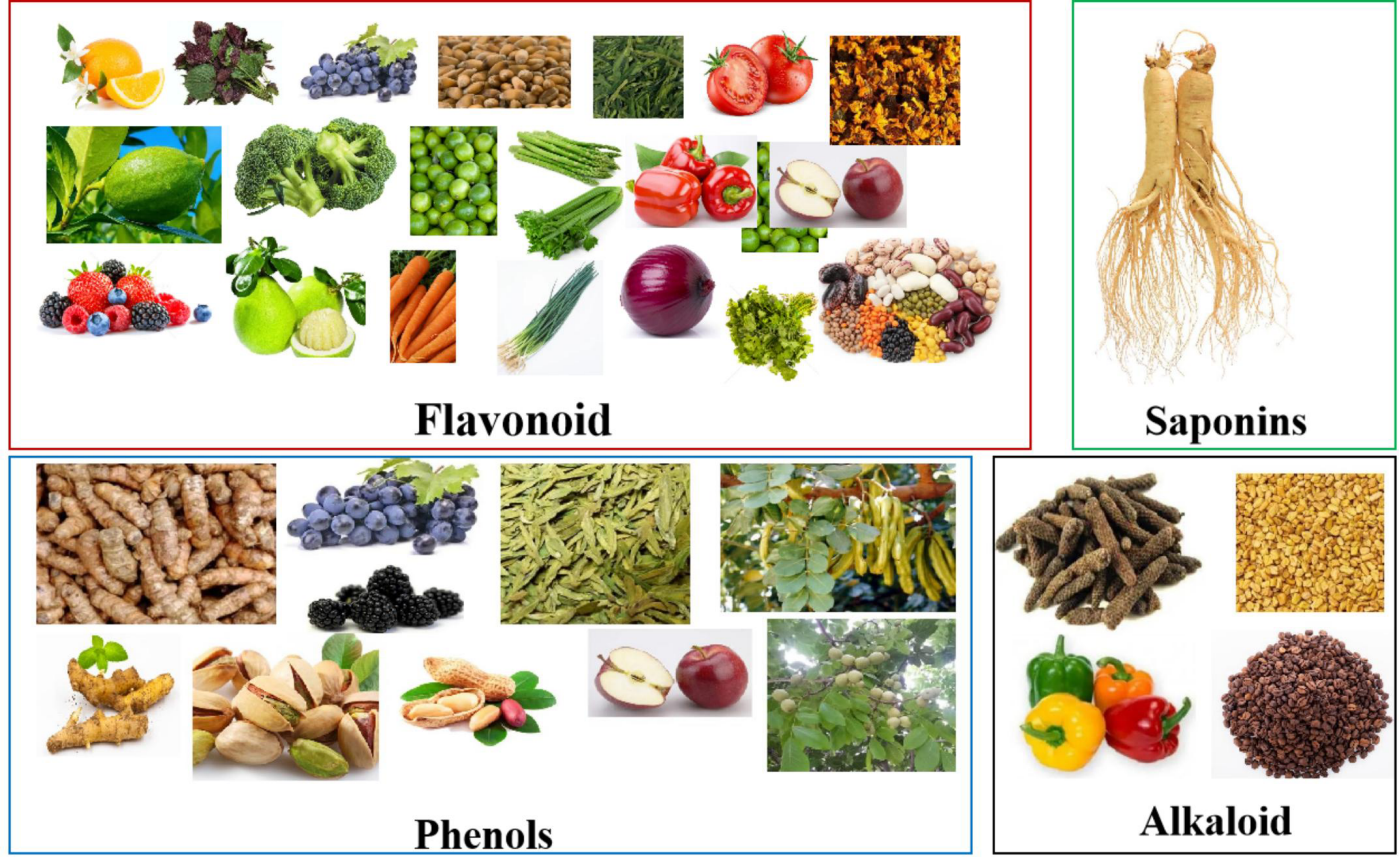
Food sources of dietary compounds that induce ferroptosis.

Dietary bioactive compounds have various health-promoting activities, including anticancer, antioxidant, immune-modulating, antimicrobial, and antiparasitic effects (71, 72). Recently, extensive research has revealed foodborne compounds as ferroptosis inducers and their relevant mechanisms in cancer therapy (73). This section summarizes several dietary bioactive compounds, including saponins, flavonoids, phenols, and alkaloids that can induce ferroptosis in multisystem cancers (**Figure 2**; **Table 1**). **Table 2** summarizes the tumor-suppressive concentration ranges of food-derived compounds, the types of cancers they affect, and a comparison of the mechanisms between food-derived compounds and synthetic ferroptosis inducers. These food-derived bioactive compounds are abundant in fruits, vegetables, grains, seeds, and spices (**Figure 3**).

**Table 1:** Inhibition and mechanisms of multisystem tumors by food-derived compounds as ferroptosis inducers.

Classification	Compounds	Food sources	Test model	Does administered	Mechanisms of inducing ferroptosis	Authors (Reference)
Saponins	Ginsenoside Rh3	ginseng	HT29 and HCT116 cells; colorectal cancer xenograft model	20, 40 and 80 μM for 48 h; 20 mg/kg/d for 21 days	Through the Stat3/p53/NRF2 axis	Wu et al. (74)
	Ginsenoside Rh4	ginseng	HT29 and HCT116 cells; colorectal cancer xenograft model	50, 100 and 200 μM for 48 h; 40 mg/kg/d for 21days	Through autophagy	Wu et al. (75)
	Ginsenoside Rh4	ginseng	Myeloma NCI-H929 cells	25, 50, 100 and 200 μM for 24h or 48h	Regulating SIRT2	Ying et al. (76)
	Ginsenoside Rh4	ginseng	Human RCC cell lines ACHN and 786-O	100 µM for 24 h	Through the NRF2 pathway	Zhao et al. (77)
	Ginsenoside Rg5	ginseng	GSCh02 and GSCh05 cells; glioblastoma xenograft model	0-1280 nM for 24h; 2 μ(0, 25 and 50 μM) for 60 days	Via NR3C1/HSPB1/NCOA4 axis	Zhang et al. (78)
	Ginsenoside CK	ginseng	HepG2 SK-Hep-1 QSG-7701; liver cancer xenograft model	20, 40 and 60 µM for 7 days	Via the FOXO pathway, expression levels of GPX4 and SLC7A11 in cells↓.	Chen et al. (79)
	Ginsenoside RK1	ginseng	liver cancer HepG2 and Hep3B cells	10, 20 and 40 μM for 24 h.	Through an FSP1-dependent pathway	Jiang et al. (80)
Flavonoid	Eriodictyol	fruits or vegetables	CaoV3 and A2780 cells; HCC xenograft model	800 μM for 24h and 48 h; 100 mg/kg for 1 month	In vitro, Fe2+ and ROS↑; SLC7A11 and GPX4↓; In vivo, via the Nrf2/HO-1/ NQO1 signaling pathway	Wang et al. (81)
	Naringenin	Grapefruit, oranges, tomatoes	HepG2, Hep3B and SNU182 cell lines; liver cancer xenograft model	0.2 mM and 0.4 mM for 24h ;50 mg/kg 14 days	The inhibition of aerobic glycolysis mediated by the AMPK-PGC1α signaling pathway	Li et al. (82)
	Hesperidin	lime peel powder	HNSCC xenograft model	50 mg/kg/d for 5 days	As a TRIB3 inhibitor, inhibit TRIB3 interacts with β-catenin and TCF4, promoting the transcriptional activity of ALOXE3	Chen et al. (83)
	Hesperetin	tangerines, oranges and grapefruit	bladder cancer T24(HTB-4) and 5637(HTB-9) cells	0-800µM for 24 h and 48 h	Targeting proteins such as SRC, PIK3R1, and MAPK1, as well as the PI3K/AKT pathway, activate ROS and decrease GPX4 expression	Lv et al. (84)
	Apigenin	fruits, vegetables, wheat sprouts and some seasonings	Myeloma NCI-H929 cells	IC50 value: 10.73 ± 3.21 μM for 72h	Inhibition of the STAT1/COX-2/iNOS signaling pathway	Adham et al. (85)
	Quercetin	apples, asparagus, berries, onions, red wine, tea, beans and tomatoes	liver cancer HepG2, Hep3B and MDA-MB-231; colorectal cancer HCT116	50µM for 24h	Induces EB-mediated lysosomal activation, increases ferritin degradation	Wang et al. (86)
	Quercetin	apples, asparagus, berries, onions, red wine, tea, beans and tomatoes	AGS, HGC-27, MKN-7, MKN-45, SNU-1 and NCI–N87; gastric cancer xenograft model	40µM for 24h; 20 mg/kg/d for 28 days	Targeting SLC1A5 and suppressing the NRF2/xCT pathway; activating the p-Camk2/p-DRP1 pathway, and accelerates iron accumulation.	Ding et al. (87)
	Quercetin	apples, asparagus, berries, onions, red wine, tea, beans and tomatoes	AGS, MKN45, MKN7 and TMK1; gastric cancer xenograft model	160 µM for 48h; 20mg/kg/d for 4 weeks	Promoting autophagy-mediated ferroptosis	Huang et al.(109; *Qin et al. (* 88)
	Quercetin	apples, asparagus, berries, onions, red wine, tea, beans and tomatoes	Breast cancer MCF-7 and MDA-231 cells	0.1, 1 and 10 *μ*M for 24h	Promoting TFEB nuclear translocation and activates lysosomal degradation of ferritin	An et al. (89)
	Quercetin	apples, asparagus, berries, onions, red wine, tea, beans and tomatoes	LUAD CAL27 and SCC15 cells	0–150μM for 48h	Targeting proteins ALDOA and CD47	Tian et al. (90)
	Nobiletin	citrus peel	Melanoma SK-MEL-28 cells	5, 15 and 45 μM for 48 h; 15 μM for 24, 48, and 72 h.	Modulating the GSK3β-controlled Keap1/Nrf2/HO-1 signaling pathway	Feng et al. (91)
	Luteolin	celery, sweet bell peppers, carrots, onion leaves, broccoli, and parsley	HCT116 and SW480 cells; colon cancer xenograft model	1.56, 3.125, 6.25, 12.5, 25, and 50 μM for 24h; 60 mg/kg every 2 days for eight times	Through HIC1-mediated suppression of GPX4 expression	Zheng et al. (92)
	Luteolin	celery, sweet bell peppers, carrots, onion leaves, broccoli, and parsley	DU145 and PC-3 cells; prostate cancer xenograft model	60 μM for12, 24, and 48 h ;5 mg/kg, once every 3 days for a total of 21 days	Promoting TFEB nuclear translocation and increasing ferritinophagy	Fu et al. (93)
	Luteolin	celery, sweet bell peppers, carrots, onion leaves, broccoli, and parsley	nasopharyngeal carcinoma NPC53 and HNE3 eells	30μM for 48h	Inhibiting SOX4 expression to reduce the binding of SOX4 to the GDF15 promoter, downregulating GDF15 transcription and inducing ferroptosis	Wu et al. (94)
	Luteolin	celery, sweet bell peppers, carrots, onion leaves, broccoli, and parsley	786-O and OS-RC-2 cells; ccRCC xenograft model	40 and 60 *μ*M for 24h or 48h; 50 mg/kg/d for 7 days	heme degradation and LIP↑; HO-1 and Fenton reaction↑; GSH↓, LPO↑	Han et al. (95)
Phenols	Curcumin	turmeric (*Curcuma longa*)	A549 and H1299 cells; Lung cancer xenograft model	0-40 *μ*M for12, 24 and 48h ; 100 mg/kg/d for 15 days	Via activating autophagy	Tang et al. (96)
	Curcumin	turmeric (*Curcuma longa*)	LK-2 and H1650; NSCLC xenograft model	10, 20 and 40 μM for 24 h; 50 mg/kg/d for 4 weeks	Regulating DMRT3/SLC7A11 axis	Xu et al. (97)
	Curcumin Analog, HO-3867	turmeric (*Curcuma longa*)	NSCLC H460, PC-9, H1975, A549 and H1299 cells	5, 10, 20, 40, and 80 *μ*M for 24 h, 48 h, and 72 h	Through the activation of the p53-DMT1 pathway and inhibition of GPX4	Wu et al. (98)
	Curcumin	turmeric (*Curcuma longa*)	Lung cancer A549 CD133+ cells	0, 10, 20, 40, 80 μM for 24h	Inhibiting the GSH-GPX4 and FSP1-CoQ10-NADH pathways	Zhou et al. (99)
	Curcumin	turmeric (*Curcuma longa*)	MNNG/HOS and MG-63; osteosarcoma xenograft model	22.5 μM for 24, 48 and 72h; 50mg/kg/d for 4weeks	regulating the Nrf2/GPX4 signaling pathway	Yuan et al. (100)
	EF24(a synthetic analogue of curcumin)	turmeric (*Curcuma longa*)	Osteosarcoma U2os cells	0.5, 1, 2 , 4 μM for 24h	Upregulated HMOX1 to suppress GPX4 expression; MDA, ROS and intracellular ferric ion level↑.	Lin et al. (101)
	Curcumin	turmeric (*Curcuma longa*)	Colorectal cancer SW480 and HCT116	IC50s value: 3.0 μg/mL in SW480 and 2.4 μg/mL in HCT116 for 24h	Inhibition of PI3K/Akt/mTOR signaling pathway	Chen et al. (102)
	Curcumin	turmeric (*Curcuma longa*)	Colorectal cancer SW-480 and HTH-29 cells	The IC50 value: 1.7 μM in SW-480 and 29.2 μM in HT-29 cells	Dual Suppression of GPX4 and FSP1	Miyazaki et al. (103)
	Curcumin	turmeric (*Curcuma longa*)	SW620 cells and LoVo cells; CRC xenograft tumors model	10, 20 and 40 μM for 0, 12, 24 h; 50, 100, and 200 mg/kg/d for 16 days	Downregulation of JNK signaling	Xin et al. (104)
	Curcumin	turmeric (*Curcuma longa*)	CRC SW620 cells and LoVo cells	IC50 values of 24, 48h and 72h: SW620 cells 30.54, 24.68, and 21.86 μM; LoVo cells, 40.22, 27.58, and 21.14 μM.	Regulation of p53 and SLC7A11/glutathione/GPX4 axis	Ming et al. (105)
	Curcumin	turmeric (*Curcuma longa*)	human GC cells (AGS and HGC-27)	0, 10, and 20 μM for 24 or 48 h.	Inhibition of the PI3K/Akt/mTOR signaling pathway	Zheng et al. (106)
	Curcumin	turmeric (*Curcuma longa*)	MDA-MB-453 and MCF-7; breast cancer xenograft model	20 μM for 48 h; 30 mg/kg/d for 4 weeks	Promoting SLC1A5-mediated ferroptosis	Cao et al. (107)
	Curcumin	turmeric (*Curcuma longa*)	MCF-7 and MDA-MB-231 cells; breast cancer xenograft model	IC50 values: 41.90 *μ*M in MCF-7 and 53.51 *μ*M in MDA -MB-231 for 48h	Inducing HO-1	Li et al. (108)
	Curcumin	turmeric (*Curcuma longa*)	Breast cancer xenograft model	5–50 µM for 48h	Inducing HO-1	Consoli et al. (109)
	Curcumin	turmeric (*Curcuma longa*)	Thyroid cancer FTC-133 and FTC-238 cells	IC50 values: 23.29 *μ*M in FTC-133 and 22.62 *μ*M in FTC -238 cells for 24h	Inducing HO-1	Chen et al. (110)
	Curcumin	turmeric (*Curcuma longa*)	ccRCC A498-DR and 786-O-DR cells	0-10um for 48h	Upregulation of the *ADAMTS18* gene	Xu et al. (111)
	curcumin derivative NL01	turmeric (*Curcuma longa*)	Ovarian cancer Anglne and HO8910PM cells	1, 2, 4, and 8 μM for 4 days.	Via the SREBP1 pathway	Shi et al. (112)
	curcumin derivative, MitoCur-1	turmeric (*Curcuma longa*)	Human colorectal cancer cell lines HT29 and HCT116 cell lines	0, 1, 2, and 4 μM for 12h	Inhibits USP14, inactivates the GPX4, while increasing GSH depletion and reducing SLC7A11 expression levels	Li et al. (113)
	Resveratrol	Peanuts, pistachios, bilberries and blueberries	CRC HT29 and HCT116 cells	The IC50 values: 47.85±0.96 µg/ml in HT29 and 23.65±3.21 µg/ml HCT116 cells	ROS and LPO↑; SLC7A11 and GPX4↓	Zhang et al. (114)
	Resveratrol	Peanuts, pistachios, bilberries and blueberries	CRC HT29 xenograft model	10 mg/kg/d for 7 days	ROS and LPO↑; SLC7A11 and GPX4↓	Jia et al. (115)
	Resveratrol	Peanuts, pistachios, bilberries and blueberries	Lung cancer H520 cells	5, 10, 25, 50, 100 μmol/L for 48h	Regulates SLC7A11-HMMR interaction to activates ferroptosis; Cancing the cytotoxic effect of CD8^+^T cells, and regulates the tumor immune microenvironment	Shan et al. (116)
	Epigallocatechin-3-Gallate	green tea leaves, apples, blackberries and carob flour	Lung cancer A549 and H1299 cells	0, 20, 40, 60, 80, and 100 µM for 72 h	Downregulating the key target tsRNA-13502 and GPX4, SLC7A11 and ACSL4; accumulation of iron, MDA, and ROS↑	Wang et al. (117)
	Epigallocatechin-3-Gallate	green tea leaves, apples, blackberries and carob flour	A549 cells; lung cancer xenograft model	20µM for 24 h; 1% EGCG for 16 weeks	Via STAT1/SLC7A11 pathway and gut microbiota	Li et al. (118)
	6-Gingerol	*Zingiber officinale*	Prostate cancer LNCaP, DU145 and PC3 cells	1–500 µM for 24, 48 or 72 h.	GPX4 and Nrf2↓; ROS↑	Liu et al. (119)
	6-Gingerol	*Zingiber officinale*	A549 cell; lung cancer xenograft model	20, 40, 80 μM for 24h; 0.25 mg/kg/d for 20 days	Inhibition of USP14 enhances autophagy- dependent ferroptosis	Tsai et al. (120)
	6-shogaol	*Zingiber officinale*	EC Ishikawa cells	0, 20, 25 μM, and 30 μM for 24h; 50 mg/kg/d for 16 days	PI3K/AKT pathway	Ma et al. (121)
	Juglone	fresh ripe fruit husk, roots, leaves, and bark of walnut trees	lung cancer A549 cells	10–50 µM for 24h	HMOX1 activation; iron overload; xCT inhibition; GSH and GPX4↓; ROS and LPO↑	Du et al. (122)
	Juglone	fresh ripe fruit husk, roots, leaves, and bark of walnut trees	Pancreatic cancer MIA Paca-2 cells	0.5-20 μM for 24 h	Induces cell death can be reversed by deferoxamine	Karki et al. (123)
	Juglone	fresh ripe fruit husk, roots, leaves, and bark of walnut trees	EC Ishikawa cells	10, 15 and 20 μM for 24 h	Fe2+ overload; LPO↑; GSH↓; HMOX1↑, and the degradation of heme into Fe2+↑	Zhang et al. (124)
Alkaloid	Piperlongumine	edible long piper plants	oral squamous carcinoma HSC-3 cells H400 cells	IC50 values: for HSC-3 cells, 14.57 μM (24 h) and 4.02 μM (48 h); For the H400 cells, 28.34 μM (24 h) and 14.71 μM (48 h)	LPO and intracellular ROS↑; DMT1↑; FTH1, SLC7A11, and GPX4↓	Wang et al. (125)
	Piperlongumine	edible long piper plants	Lung cancer MCF-7 and A549 cells	100 µM for 30 min	inhibiting TXNRD1	Yang et al. (126)
	Piperlongumine	edible long piper plants	Pancreatic cancer PANC-1 cell	14 *μ*M for 16h	intracellular ROS↑; GSH↓, which was blocked by ferroptosis inhibitors and iron chelators	Yamaguchi et al. (127)
	Trigonelline	coffee beans and fenugreek seeds	head and neck cancer cells; mice xenograft models	0.15 or 0.3 mM for 6h;50 mg/kg/d for 20 days	inhibiting the Nrf2 system	Roh et al. (128); Shin et al. (129)
	Trigonelline	coffee beans and fenugreek seeds	HCC xenograft model	1 mg/kg, once every other day for two weeks	Inhibited the expression of the Nrf2 target genes NQO1, HO-1, and FTH1; Suppressing Nrf2 activation	Sun et al. (130)
	Capsaicin	genus *Capsicum*	NSCLC A549 and NCI-H23 cells	IC50 value: 200 μM(48 h) for A549 cells and 100 μM(48 h ) for NCI-H23 cells	Inactivating SLC7A11/GPX4 signaling	Liu et al. (131)
	Capsaicin	genus *Capsicum*	Glioblastoma U87-MG and U251 cells	IC50 value: 121.6 μM(24 h) for U87-MG cells and 237.2 μM(24 h) for U251 cells	Through ACSL4/GPx4 signaling pathways	Hacioglu et al. (132)

**Table 2:** The tumor-suppressive concentration range of dietary compounds and its comparison with synthetic ferroptosis inducers.

Compounds	Cancer kinds	Cells	Tumor suppression concentration range	Comparison with synthetic ferroptosis inducers	Authors (Reference)
Ginsenoside GRh3	CRC	HT29, HCT116, RKO, SW620, and DLD1 cells	0-160µM		Wu et al. (74)
Ginsenoside Rh4	CRC	HT29, HCT116, DLD1, and RKO cells	0-300 µM		Wu et al. (75)
	Multiple myeloma	ACHN and 786-O	100 µM	Rh4 Enhanced the Sensitivity of RCC Cells to RSL3- Induced Ferroptosis	Zhao et al. (77)
	Glioblastoma	GSCh02 and GSCh05	0-1280 µM		Zhang et al. (78)
Ginsenoside Rg5	Glioblastoma	GSCh02 and GSCh05)	0-1280 µM		Zhang et al. (78)
Ginsenoside CK	Liver cancer	HepG2 and SK-Hep-1	20-60 µM		Chen et al. (79)
Eriodictyol	Ovarian cancer	CaoV3 and A2780	0-800 µM		Wang et al. (81)
Naringenin	Liver cancer	HepG2, Hep3B and SNU182 cell lines	0.2-0.4mM	Naringenin enhances the sensitivity of liver cancer cells to erastin- and RSL3-induced ferroptosis, the synergistic effect between NAR and ferroptosis inducers	Li et al. (82)
Hesperetin	Bladder cancer	T24(HTB-4) and 5637(HTB-9) cells	0- 800 µM		Lv et al. (84)
Quercetin	Human HCC	HepG2 , Hep3B, MDA-MB-231	12.5-100 µM		Wang et al. (86)
	CRC	HCT116	12.5-100 µM		Wang et al. (86)
	Gastric cancer	AGS, HGC-27, MKN-7, MKN-45, SNU-1 and NCI–N87;	0-200 µM		Ding et al. (87)
	Breast cancer	MCF-7 and MDA-231 cells	0.1- 10 µM		An et al. (89)
	LUAD	CAL27 and SCC15 cells	0–150 μM		Tian et al. (90)
Luteolin	Colon cancer	HCT116 and SW480 cells	1.56-50 µM		Zheng et al. (92)
	Prostate cancer	DU145, PC-3, VCaP and LNcaP	0- 60 μM		Fu et al. (93)
	nasopharyngeal carcinoma	NPC53 and HNE3 eells	15-60 µM		Wu et al. (94)
	ccRCC	786-O and OS-RC-2 cells	0- 128 µM		Han et al. (95)
Curcumin	Lung cancer	A549 and H1299 cells	0-40 µM		Tang et al. (96)
	NSCLC	LK-2 and H1650	10- 40 μM		Xu et al. (97)
	Lung cancer	A549 CD133+ cells	0- 80 μM	Curcumin, RSL3 respectively suppressed the GSH /GPX4 and FSP1/CoQ10 /NADH pathways in A549 CD133+ cells.	Zhou et al. (99)
	Osteosarcoma	MNNG/HOS and MG-63	12-36 µM		Yuan et al. (100)
	CRC	SW-480 and HTH-29 cells	1-5 µg/mL		Chen et al. (102)
	CRC	SW620 cells and LoVo cells	10- 40 μM		Xin et al. (104)
	CRC	SW620 cells and LoVo cells	10- 80 μM		Ming et al. (105)
	Gastric cancer	human GC cells (AGS and HGC-27)	0- 20 μM		Zheng et al. (106)
	Breast cancer	MDA-MB-453 and MCF-7 cells	0- 50 μM.		Cao et al. (107)
	Breast cancer	MCF-7 and TNBC MDA-MB 231	5–50 µM	At a concentration of 50 μM, both Curcumin and erastin effectively reduce GPX4 levels. After 24 hours of erastin treatment, FHC levels showed no decrease; in contrast, Curcumin treatment had a significant effect.	Consoli et al. (109)
	Thyroid cancer	FTC-133 and FTC-238 cells	0- 20 µM	Erastin, like curcumin, can both promote the expression of HO-1 and activate ferroptosis in FTC cells.	Chen et al. (110)
	ccRCC	A498-DR and 786-O-DR cells	0-10 µM		Xu et al. (111)
Resveratrol	Lung cancer	H520 cells	5- 100 μmol/L		Shan et al. (116)
Epigallocatechin-3-Gallate	Lung cancer	A549 and H1299 cells	0- 100 µM		Wang et al. (117)
6-Gingerol	Prostate cancer	LNCaP, DU145 and PC3 cells	1–500 µM		Liu et al. (119)
6-Gingerol	Prostate cancer	LNCaP, DU145 and PC3 cells	1–500 µM		Liu et al. (119)
6-shogaol	EC	Ishikawa cells	0- 30 μM		Ma et al. (121)
Juglone	lung cancer	A549 cells	10–50 µM		Du et al. (122)
	Pancreatic cancer	MIA Paca-2, BXPC-3, and Panc-1	0.5-20 μM		Karki et al. (123)
	EC	Ishikawa cells	10- 20 μM		Zhang et al. (124)
Piperlongumine	Oral squamous carcinoma	H400 and HSC-3	0-50 μM		
	Lung cancer	MCF-7 and A549	0.1-50 μM	Low concentrations of piperlongumine do not induce ferroptosis, but piperlongumine enhances erastin-induced cell death in cancer cells by inhibiting the activity of TXNRD1.	Wang et al. (125)
	Pancreatic cancer	MIAPaCa-2, PANC-1, CFPAC-1, and BxPC-3	0-14 μM	The triple combination treatment using Piperlongumine, Cotylenin A (CN-A, a plant growth regulator), and sulfasalazine (SSZ, a ferroptosis inducer) is highly effective in treating pancreatic cancer.	Yamaguchi et al. (127)
Trigonelline	HNC and SNU	AMC-HN2–11 and SNU-1041, −1066, and −1076	0.15mM, 0.3mM	Trigonelline sensitized chemoresistant HNC cells to RSL3 treatment	Shin et al. (129)

### 3.1 Saponins

Saponins have been applied in the treatment of various diseases for many years in clinical practice.

**Figure 4:**
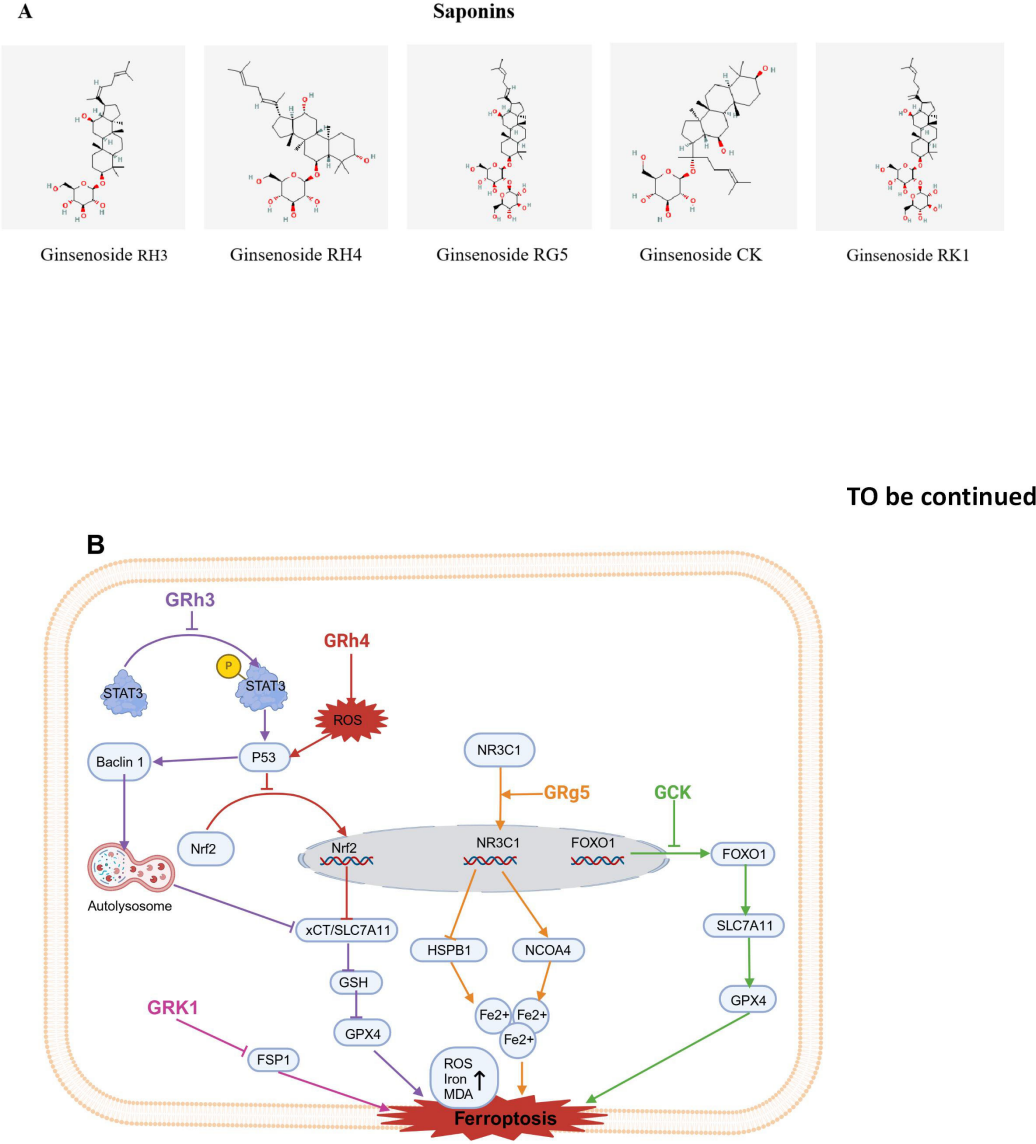
Chemical structures of food-derived saponins and the mechanism by which ferroptosis is induced to inhibit tumours. **(A)** Chemical structures of food-derived saponins. **(B)** The mechanism by which food- derived saponins induce ferroptosis to inhibit tumours. The map was created using biorender.

In this section, a total of 5 flavonoids, i.e., Ginsenoside Rh3, Ginsenoside Rh4, Ginsenoside Rg5, Ginsenoside CK,Ginsenoside CK,, which are potential inducers of ferroptosis are reviewed. The molecular structures of food-derived flavonoid compounds used as inducers of ferroptosis are shown in **Figure 4A**. The mechanism by which food-derived flavonoidcompounds induce ferroptosis to inhibit tumors is detailed in **Figure 4B**.

#### 3.1.1 Ginsenoside Rh3

Ginsenoside Rh3 (GRh3) is a semi-synthetic triterpenoid compound known for its strong anticancer properties (133); it is a bacterial metabolite of ginsenoside Rg5, the main component of hot-processed ginseng (134). GRh3 effectively suppressescolorectal cancer cells proliferation. GRh3 prevents NRF2 from entering the nucleus, which in turn inhibits SLC7A11.This causes a depletion of GSH, an accumulation of iron, and elevated levels of ROS and MDA, ultimately triggering ferroptosis in colorectal cancer cells (74). Subsequently, studies revealed that p53 inhibitor obviously decreased P53 protein expression induced by GRh3 as well as NRF2 nuclear translocation; additionally, changes in ferroptosis-related proteins induced by GRh3 were reversed. However, Stat3 phosphorylation was not reversed by pretreatment with the p53 inhibitor. Overall, GRh3 promotes ferroptosis through the Stat3/p53/NRF2 axis, demonstrating significant anticancer potential in colorectal cancer cells (74).

#### 3.1.2 Ginsenoside Rh4

Ginsenoside Rh4 (Rh4) is a natural dammarane glycoside, derived from Korean ginseng (135). Rh4 has been found to have antitumor activity via suppressing migration and proliferation of cancer cells (136, 137). Research by Wu et al. revealed that Rh4 inhibited the growth of CRC xenograft tumors, causing only minimal side effects. Further mechanistic studies revealed that Rh4 significantly upregulated the expression level of autophagy markers, such as protein p53 and protein Beclin1and ferroptosis markers (proteins xCT/SLC7A11 and GPX4) *in vivo* and *in vitro*. Interestingly, Rh4-induced ferroptosis was reversed by ferroptosis inhibitor ferrostatin-1 (Fer-1) and the autophagy inhibitor 3-methyladenine (3-MA), which suggest that Rh4-induced ferroptosis is regulated through the autophagy pathway. Additionally, Rh4 elevated ROS accumulation, which activatedROS/p53 signaling pathway, thus demonstrating that Rh4 inhibits proliferation of cancer cell via promoting the ROS/p53 signaling pathway and inducing ferroptosis through autophagy (75).

In addition, Ying and others reported that Rh4 inhibited the proliferation of multiple myeloma (MM) cells. Further mechanistic studies revealed that Rh4 also inhibited the expression of SIRT2. SIRT2 overexpression counteracted the impact of Rh4 on MM cell proliferation, apoptosis, cell cycle arrest, and ferroptosis. Thus, Rh4 induces ferroptosis by regulating SIRT2 and inhibits the progression of multiple myeloma (76).

Furthermore, Zhao et al. research indicates that Rh4 increases the sensitivity of RCC cells to RSL3-induced ferroptosis, enhancing the effect of RSL3 in inhibiting renal cell carcinoma (RCC) cells. Rh4 downregulated the expression of ferroptosis-related genes such as superoxide dismutase 1 (SOD1), GPX4, and catalase (CAT), and these effects were weakened after NRF2 knockdown. The findings suggest that Rh4 enhances ferroptosis sensitivity through the NRF2 pathway (77).

#### 3.1.3 Ginsenoside Rg5

During the ginseng steaming process, the deglucosylation reaction of ginsenoside Rb1 and the dehydration reaction of ginsenoside Rg3 at the 20th carbon position form ginsenoside Rg5 (Rg5), which is a trace ginsenoside (138). Recent research has shown that Rg5 inhibits glioblastoma progression by activating nuclear receptor subfamily 3 group C member 1 (NR3C1), thereby regulating heat shock protein family B member 1 (HSPB1) and nuclear receptor coactivator 4 (NCOA4) (78).

#### 3.1.4 Ginsenoside CK

Ginsenoside CK (CK) is an active component of ginseng. CK inhibits the proliferation of

human hepatoma HepG2 and growth of HepG2 cell transplant tumors, as well as the growth of SK-Hep-1 cells. Subsequent mechanistic studies revealed that phosphorylated FOXO1 relocates from the nucleus to the cytoplasm, resulting in a decrease in its transcriptional activity. CK was shown to inhibit the phosphorylation of FOXO1 in cells and activate the FOXO signaling pathway, significantly reducing GPX4 and SLC7A11 expression in cells. These findings indicate that CK promotes ferroptosis by blocking FOXO1 phosphorylation and activating the FOXO signaling pathway, thereby exerting antitumor effects in liver cancer cells (79).

#### 3.1.5 Ginsenoside RK1

Ginsenoside RK1 (RK1) is a rare bioactive compound extracted from ginseng through deglycosylation. RK1 exhibits a variety of biological activities, such as antiviral effects, inhibition of inflammatory responses, and tumor suppression (139). It was found that RK1 had an inhibitory effect on liver cancer cells, simultaneously reducing GSH levels while raising MDA and iron levels. Importantly, the RK1- induce d cell death was specifically blocked by Fer-1 and liprostatin-1 (Lip-1). Additionally, overexpression or silencing of FSP1 promoted or inhibited RK1-induced ferroptosis, respectively. These findings suggest that RK1 promotes ferroptosis in liver cancer via an FSP1-dependent pathway (80).

It is worth noting that ginsenoside Rg3 has already been applied in the clinical treatment of non-small cell lung cancer (NSCLC). Immune suppression caused by tumor cells and the toxic side effects of chemotherapy drugs lead to a decline in immune function, ultimately resulting in chemotherapy treatment failure. Clinical studies have shown that ginsenoside Rg3 plays a positive role in enhancing the immune function of patients. Gao et al. conducted a meta-analysis of 12 trials with a total sample size of 1008 NSCLC patients. The results found that the combined use of ginsenoside Rg3 and first-line chemotherapy better improved CD3+ T lymphocyte levels (P < 0.00001), CD4+ T lymphocytes (P < 0.00001), CD8+ T lymphocytes (P = 0.003), CD4+/CD8+ T lymphocyte ratio (P = 0.0006), increased natural killer cell activity (P = 0.007), restored chemotherapy-induced leukopenia, and improved the clinical efficacy of the patients (140).

### 3.2 Flavonoids

**Figure 5:**
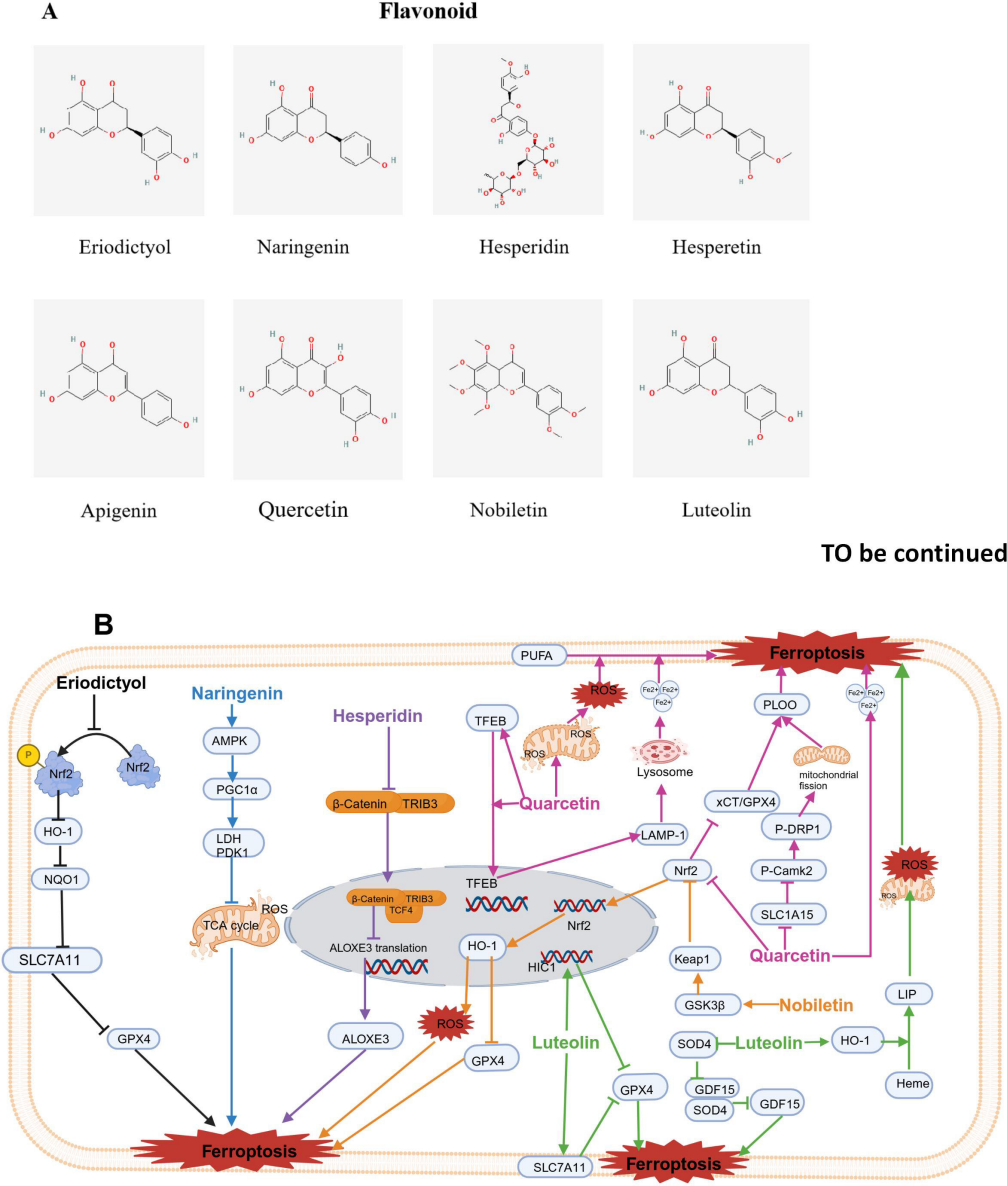
Chemical structures of food-derived flavonoids and the mechanism by which ferroptosis is induced to inhibit tumours. **(A)** Chemical structures of food-derived flavonoids. **(B)** The mechanism by which food-derived flavonoids induce ferroptosis to inhibit tumours. The map was created using biorender.

A high intake of vegetables and fruits rich in flavonoids seems to reduce the incidence of cancer, suggesting that flavonoid compounds have excellent anti-cancer potential (141). In this section, a total of 9 flavonoids, i.e., eriodictyol (EDT), naringenin (NAR), hesperidin (HSD), apigenin (API), epigallocatechin-3-gallate (EGCG), quercetin (QUE), erastin (ERT), nobiletin (NOB), and luteolin (LT), which are potential inducers of ferroptosis are reviewed. The molecular structures of food-derived flavonoid compounds used as inducers of ferroptosis are shown in **Figure 5A**. The mechanism by which food-derived flavonoid compounds induce ferroptosis to inhibit tumors is detailed in **Figure 5B**; **Table 1**.

#### 3.2.1 Eriodictyol

Eriodictyol (EDT) is a kind of flavonoid that is abundantly extracted from plants, fruits and vegetables (142). Previous studies have shown that EDT inhibited the activity of the ovarian cancer cell lines CaoV3 and A2780. Additionally, EDT increased the levels of Fe^2+^ and the production of ROS while lowering the protein levels of SLC7A11 and GPX4. Furthermore, EDT decreased the dye JC-1 fluorescence ratio, GHS, and MDA content but increased the level of cytochrome C. Nrf2 phosphorylation was significantly decreased. *In vivo* experiments revealed that EDT inhibited tumor growth, exacerbated mitochondrial dysfunction, and decreased Nrf2 expression in mouse tumor tissues. These results indicate that EDT inhibits the proliferation of ovarian cancer cells and is closely associated with the regulation of ferroptosis, mitochondrial dysfunction, and cell viability through the Nrf2/HO-1/NQO1 signaling pathway (81).

#### 3.2.2 Naringenin

Naringenin (NAR) is a flavanone and that is found in large amounts in foods such as grapefruit, oranges, and tomatoes (143). NAR has been used in cosmetic, perfumery, and pharmaceutical products. According to the results of preclinical studies, NAR has a wide range of biological and pharmacological effects involving anticarcinogenic properties (144, 145).

Li et al. explored the synergistic effect of NAR and ferroptosis inducers using liver cancer cell lines and xenograft mice. The results show that when non-toxic doses of NAR were used in combination with ferroptosis inducers like erastin, RSL3, and sorafenib, NAR significantly enhanced the anticancer effects of the ferroptosis inducers. The combination index method confirmed that there was a synergistic antitumor effect between NAR and the ferroptosis inducers. Compared with NAR or ferroptosis inducers alone, the combined treatment resulted in greater lipid peroxidation, resulting in increased ferroptosis damage to cancer cells. The synergistic cytotoxic effects of NAR and ferroptosis inducers were reversed when the cells were pretreated with AMPK inhibitors or PGC1α inhibitors, confirming that the inhibition of aerobic glycolysis mediated through the AMPK-PGC1α signaling pathway is crucial for NAR reducing liver cancer resistance to ferroptosis (82).

#### 3.2.3 Hesperidin and hesperetin

Hesperidin (HSD) is a subgroup of citrus flavonoids, primarily extracted from bitter orange peel powder (146). Chen and colleagues reported that tribble protein kinase 3 (TRIB3) promoted head and neck squamous cell carcinoma (HNSCC) progression by weakening ferroptosis. In addition, mechanistically, TRIB3 interacted with β-catenin and TCF4 to form a trimeric complex, thereby inhibiting the transcriptional activity of ALOXE3 and subsequently suppressing ferroptosis (83).

Hesperetin (HE) is primarily found in citrus fruits like tangerines, oranges, and grapefruit. Recently, it has gained attention for its potential antitumor effects against various types of cancer (147). Through network pharmacology prediction and experimental validation, the key targets of HE in bladder cancer (BLCA) include SRC, PIK3R1, and MAPK1, primarily affecting the PI3K/AKT pathway. HE was shown to activate ROS and decrease GPX4 expression, suggesting that HE may induce ferroptosis and inhibit BLCA processes (84).

#### 3.2.4 Apigenin

Apigenin (API) is widely found in various plant-based foods, such as fruits, vegetables, wheat sprouts, and seasonings. It is one of the most abundant flavonoid compounds in nature (148). A study by Adham confirmed that ferroptosis played a role in API-induced cell death. The impact of API on NCI-H929 cells decreased by over three times when the cells were coincubated with ferroptosis inhibitors like the iron chelators Fer-1 and deferoxamine, suggesting that apigenin induces the death of NCI-H929 cells through ferroptosis (85).

#### 3.2.5 Quercetin

Quercetin (QUE) is found in vegetables and fruits, such as apples, asparagus, berries, onions, and tomatoes, beans, red wine, and tea (149). Wang et al. reported that QUE increased P53-independent cell death in liver and colorectal cancer cell lines. Both lysosomal inhibitors and the knockdown of the transcription factor EB prevented cell death induced by QUE, indicating lysosomal was involved. Moreover, QUE triggered lysosomal activation by promoting the nuclear translocation of EB and activating the transcription of lysosomal genes. Notably, QUE enhanced lysosomal-dependent ferritin degradation and the release of free iron. This action synergized with QUE-induced ROS generation, leading to lipid peroxidation and ferroptosis. Additionally, Bid may link ferroptosis and apoptosis, resulting in cell death. These findings suggest that QUE induces EB-mediated lysosomal activation, increases ferritin degradation, and leads to ferroptosis and Bid-involved apoptosis (86).

Another study revealed that QUE hindered the progression of gastric cancer. Mechanistically, QUE targets SLC1A5 in gastric cancer cells, suppresses the NRF2/xCT pathway, activates the p-Camk2/p-DRP1 pathway, and accelerates iron accumulation (87). Additionally, Huang et al. indicated that QUE significantly reduced gastric cancer cell viability and tumor volume. Mechanistic studies revealed that QUE lowered the GSH, MDA and ROS contents and decreased the levels of beclin1 and LC3B in gastric cancer cells. Notably, siATG5 reversed all the aforementioned effects of QUE (150). Qin et al. showed that ATG5 plays a key role in autophagy-induced ferroptosis. Mice lacking ATG5 exhibited increased FTH1 expression and reduced ferroptosis, supporting the dependency of ferritin autophagy on ATG5 (88). These findings indicated that QUE promotes autophagy-mediated ferroptosis in gastric cancer.

QUE not only inhibited tumor growth in gastric cancer but also exerted anti-proliferative effects on breast cell lines (89) and oral squamous cell carcinoma (OSCC) (151) by inducing ferroptosis; it induced breast cancer cell death occurring in a concentration-dependent manner and increased the protein levels of iron, MDA, and carbonylated. TFEB is characterized by high expression in the nucleus and low expression in the cytoplasm. High TFEB expression increased the expression of the lysosomal gene LAMP-1, leading to the degradation of ferritin, and then releasing Fe^3+^. TFEB siRNA and chloroquine can block the pharmacological activity of QUE. QUE enhanced expression of TFEB and nuclear transcription of TFEB and induced ferroptosis, thus killing breast cancer cells (89). Additionally, QUE induced lipid peroxidation and lowered GSH levels in OSCC cells by inhibiting SLC7A11 expression, and its effects on ferroptosis and the phosphorylation of mTOR and S6KP70 were partially blocked by mTOR agonists (151). However, in Parkinson’s disease models, quercetin inhibits ferroptosis through activation of Nrf2-SLC7A11 axis, thus protecting dopaminergic neurons (152). The response to quercetin may vary across different cell types. For example, cancer cells may be more sensitive to ferroptosis, so quercetin induces ferroptosis by inhibiting SLC7A11. In contrast, neural cells may rely on the Nrf2-SLC7A11 axis to maintain antioxidant capacity, so quercetin protects cells by activating this axis. The effect of quercetin may be concentration-dependent. At low concentrations, quercetin may exert protective effects by activating the Nrf2-SLC7A11 axis, while at high concentrations, it may induce ferroptosis by inhibiting SLC7A11.

In lung adenocarcinoma (LUAD), molecular docking revealed that QUE bound to the target proteins ALDOA and CD47, suggesting that QUE binds directly to these targets, exhibiting high affinity and strong stability, thereby inhibiting these targets (90).

#### 3.2.6 Nobiletin

Nobiletin (NOB), as a polymethoxyflavonoid, which is derived from citrus peel, exhibits a range of biological activities, such as inhibiting inflammatory response, antioxidant, antidiabetic and neuroprotective effects (153). Recent research indicated that NOB had antitumor effects on melanoma cells. Further exploration of the underlying mechanism suggested that NOB enhanced GSK3β expression, activated Keap1, inhibited Nrf2/HO-1 pathway, led to iron and ROS accumulation, reduced GSH levels, and inactivated GPX4. Both increased ROS accumulation and GPX4 inactivity resulted in lipid peroxidation and subsequently induced ferroptosis (91).

#### 3.2.7 Luteolin

Luteolin (LT) is present in fruits and vegetables (154, 155). Zheng et al. suggested that combination of LT and erastin treatment effectively reduced the survival and growth of colon cancer cells, leading to lower glutathione levels and higher lipid peroxide levels. The combination treatment had significant therapeutic effects on colon cancer xenografts. Mechanistically, the combination of LT and erastin downregulates the overexpression of GPX4 in colon cancer cells. Additionally, cotreatment with LT and erastin obviously upregulated highly methylated gene 1 (HIC1) expression, a tumor suppressor. The overexpression of HIC1 markedly enhanced the inhibition of GPX4 expression and promoted ferroptosis. In contrast, silencing HIC1 weakened the inhibition of GPX4 expression, eliminating ferroptosis. Thus, LT and erastin work together to sensitize colon cancer cells to ferroptosis via suppression of GPX4 expression mediated by HIC1 (92).

Furtherly, Fu and colleagues reported that LT enhanced ferroptosis by increasing autophagy in human PCa cell lines (DU145 and PC-3). However, knocking down TFEB prevented LT from triggering the lysosomal degradation of ferritin. Furthermore, *in vivo,* LT increased ferroptosis in PCa through ferritinophagy. These results indicated that LT triggered ferroptosis in PCa cells by enhanced TFEB nuclear translocation and boosting ferritinophagy (93). In addition, Wu et al. studied the effect of the SOX4/GDF15 axis on LT-triggered ferroptosis in nasopharyngeal carcinoma cells (94). They reported that LT treatment induced ferroptosis, which was characterized by reduced cell viability; increased MDA contents and iron Fe^2+^; while decreased levels of SOD, GSH, and GPX4. LT also downregulated SOX4 expression, while SOX4 upregulated the transcription of the ferroptosis-related factor GDF15 by directly binding to its promoter. In contrast, GDF15 overexpression decreased the LT-induced ferroptosis in nasopharyngeal carcinoma cells. Thus, LT triggers ferroptosis in nasopharyngeal carcinoma cells via regulation of SOX4/GDF15 axis (94). Moreover, research has shown that LT significantly suppressed the survival of clear cell renal cell carcinoma (ccRCC), an effect that was accompanied by an excessive increase in intracellular Fe^2+^ and the abnormal depletion of GSH (95). LT also induced a mitochondrial membrane potential imbalance, classical morphological changes in mitochondrial ferroptosis, the generation of ROS, and LPO in ccRCC cells in a manner dependent on iron. Mechanistically, molecular docking suggested that LT may promote haem degradation and accumulate labile iron pool (LIP) through the excessive upregulation of HO-1 expression, causing the Fenton reaction, depletion of GSH, and LPO in ccRCC cells. Blocking this signaling pathway significantly prevented LT-triggered ccRCC cell death by inhibiting iron-induced death (95).

### 3.3 Phenols

**Figure 6:**
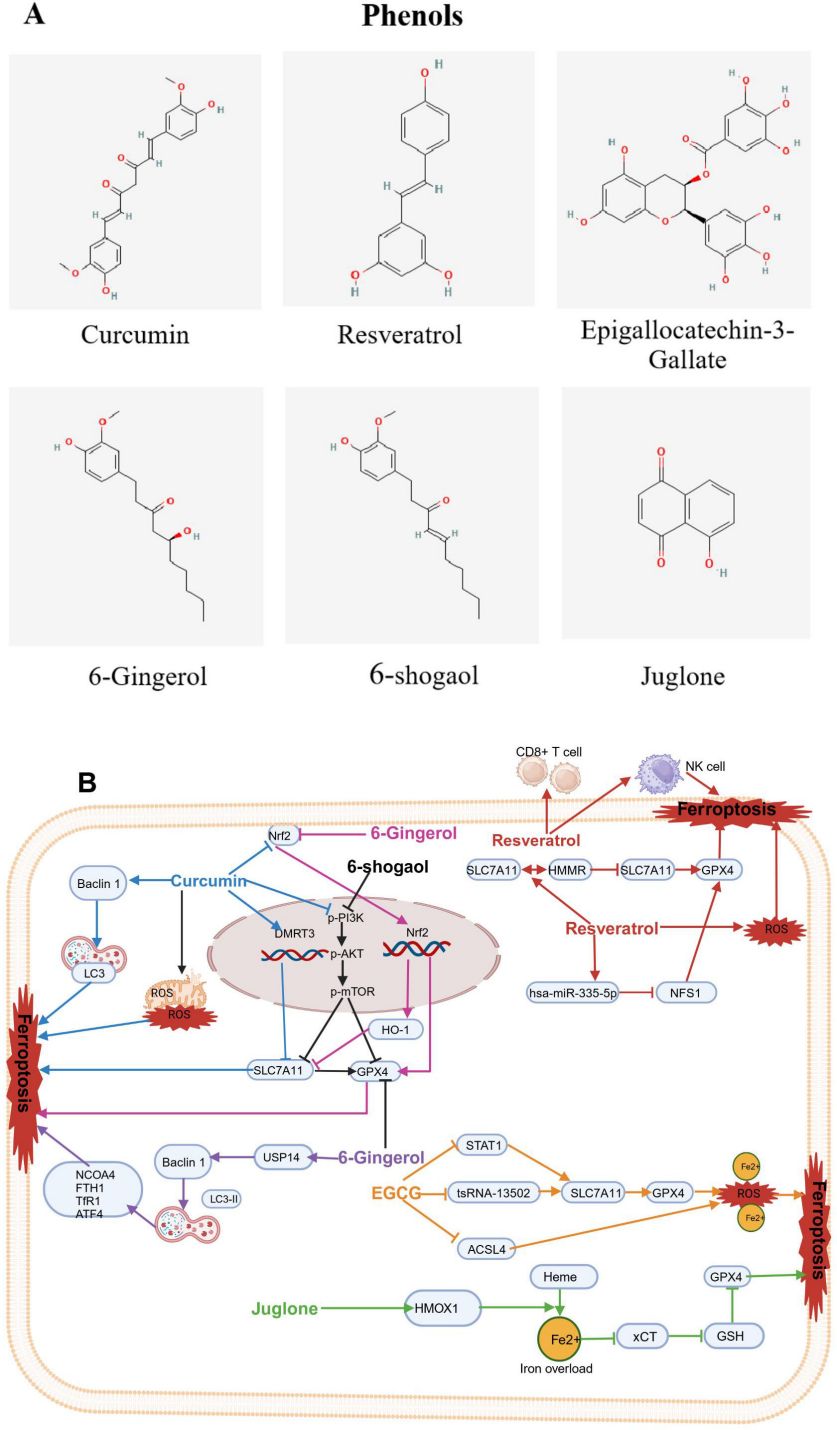
Chemical structures of food-derived phenols and the mechanism by which ferroptosis is induced to inhibit tumours. **(A)** Chemical structures of food-derived phenols. **(B)** The mechanism by which food- derived phenols induce ferroptosis to inhibit tumours. The map was created using biorender.

Various dietary phenolic compounds, such as curcumin (CUR), resveratrol (REV), epigallocatechin-3-gallate (EGCG), 6-gingerol (6G), 6-shogaol (6-SG), and juglone (JUG), have been shown to act as inducers of ferroptosis, exhibiting significant antitumor potential. The molecular structures of food-derived phenolic compounds are shown in **Figure 6A**. The mechanism by which food-derived phenolic compounds induce ferroptosis to inhibit tumors is detailed in **Figure 6B**; **Table 1**.

#### 3.3.1 Curcumin

Curcumin (CUR), as a phenolic compound, found in turmeric (Curcuma longa), has been widely used as a food additive for centuries (156), It imparts a yellowish color and unique flavor to foods. In recent decades, studies have demonstrated that curcumin possesses various biological activities, including antioxidant, anti-inflammatory, and antitumor effects (157). Recently, CUR has been shown as an ferroptosis inducer and has been used as an anticancer drug for various tumors like lung cancer, osteosarcoma, colorectal cancer, gastric cancer, breast cancer, clear cell renal cell cancer, ovarian cancer and melanoma (126-141.

Recent studies have indicated that CUR can suppressed lung cancer cells by triggering ferroptosis. Tang et al. reported that CUR inhibited proliferation of non-small cell lung cancer (NSCLC) cells. Subsequent research revealed that CUR caused mitochondrial membrane rupture, lowered the number of mitochondrial cristae, and elevated the number of autolysosomes; it increased the levels of Beclin1 and LC3 while lowering P62 levels. Both the autophagy and subsequent ferroptosis induced by CUR were alleviated by the autophagy inhibitor chloroquine (CQ) and siBeclin1 (96). Additionally, Xu B and colleagues reported that CUR inhibited NSCLC cell proliferation and angiogenesis while inducing apoptosis and triggering ferroptosis. Mechanistically, DMRT3 served as a transcription factor for SLC7A11, increasing SLC7A11 transcription by integrating with its promoter region. Moreover, CUR inhibited NSCLC growth by regulating DMRT3 *in vivo*. CUR may partly restrain the malignant phenotypes of NSCLC cells via the DMRT3/SLC7A11 axis (97). Furthermore, HO-3867, a stable CUR analogue, had strong antitumor effects on NSCLC cells, it induced ferroptosis through activating p53-DMT1 pathway and the inhibiting GPX4. Moreover, HO-3867 led to an increase in ROS in NSCLC, which dependshe accessibility of iron (98). Interestingly, CUR treatment suppressed the proliferation of A549 CD133^+^ lung CSCs (LCSCs). Mechanistically, CUR triggered ferroptosis by blocking the GSH-GPX4 pathways and FSP1-CoQ10-NADH pathways in A549 CD133^+^ cells, which led to a reduction in their self-renewal potential (99). In osteosarcoma, CUR can induce ferroptosis, therebly leading to tumor suppression. Yuan et al. revealed that CUR effectively reduced cell viability and inhibited tumor volume in an osteosarcoma xenograft model. Mechanistically, CUR triggered ferroptosis in osteosarcoma cells by regulation of the Nrf2/GPX4 signaling pathway (100). In addition, EF24, as a synthetic analogue of CUR, significantly induced cell death, elevatedHMOX1 expression, inhibited GPX4 expression, and promoted ferroptosis in osteosarcoma cell lines by elevating intracellular MDA, ROS and ferric ion levels (101).

Additionally, multiple studies have reported that CUR inhibits the proliferation of colorectal tumors by inducing ferroptosis. In terms of mechanism, CUR triggered ferroptosis and inhibited the proliferation of cancer cells via the inhibition of the PI3K/Akt/mTOR signaling pathway (102), dual suppression of GPX4 and FSP1 (103), suppression of JNK signaling (104), and regulation of P53 and the SLC7A11/GHS/GPX4 axis (105). Interestingly, CUR inhibited the proliferation of gastric cancer cells by driving ferroptosis, similar to that in colorectal cancer cells, through the inhibition of the PI3K/Akt/mTOR signaling pathway (106). Multiple studies have indicated that CUR displaysantitumorigenic activity in breast cancer (BC) by inducing ferroptosis, with mechanisms that involve promoting SLC1A5-mediated ferroptosis (107) and inducing HO-1 expression (108, 109). Furthermore, a study by Chen et al. demonstrated that the HO-1–ferroptosis pathway might exert an crucial role in follicular thyroid cancer (FTC) tumorigenesis. CUR inhibited the growth of FTC cells by increasing HO-1 expression, further activating the ferroptosis pathway (110). Xu et al. reported that combined treatment of CUR and sunitinib increased the expression of the ADAMTS18 gene and significantly decreased the expression level of NCOA4, FTH1 and p53, indicating that CUR could lower the mRNA and protein expression levels of NCOA4, FTH1, and P53, which suggested that CUR may drive ferroptosis by increasing ADAMTS18 gene expression (111). Interestingly, a Phase IIa open-label, randomized controlled trial with the registration number NCT01490996 confirmed that patients receiving folic acid/5-fluorouracil/oxaliplatin chemotherapy (FOLFOX) had a Hazard Ratio (HR) for overall survival (OS) of 0.34 (P = 0.02) when compared to the FOLFOX + 2 grams of oral curcumin/d (CUFOX). The median OS was 200 days for the FOLFOX group and 502 days for the CUFOX group. There were no significant differences between the two groups in terms of quality of life (P = 0.248) or neurotoxicity (P = 0.223). The clinical trial results suggest that curcumin, as an adjunctive treatment to FOLFOX chemotherapy, is safe and well-tolerated in patients with metastatic colorectal cancer (158). However, the issue of curcumin’s low bioavailability has garnered significant attention. Clinical trials have demonstrated that by using high concentrations of curcumin, still within non-toxic limits, the bioavailability problem of curcumin in specific situations has been addressed. Additionally, when combined with other compounds or formulations, curcumin’s bioavailability has been improved (159).

In addition to the strong antitumor effects exhibited by the CUR analogues HO-3867 and EF24 through the induction of ferroptosis, the CUR derivative NL01 has also shown promising antitumor growth characteristics in ovarian cancer cells. These effect occurred by NL01reducing HCAR1/MCT1 expression and activating the AMPK signaling pathway, thereby inducing cellular ferroptosis via the SREBP1 pathway (112). Another CUR derivative, MitoCur-1 has been shown to significantly inhibit tumor growth by triggering ferroptosis in melanoma and enhancing sensitivity to vemurafenib-resistant cells. Mechanistically, MitoCur-1 significantly inhibited USP14 and inactivated the GPX4 enzyme while exacerbating GSH depletion and reducing SLC7A11 expression levels. As a result, ferroptosis was induced by the intracellular buildup of lipid ROS, which depends on ferrous ions, thereby increasing sensitivity in vemurafenib-resistant melanoma cells (160).

#### 3.3.2 Resveratrol

Resveratrol (REV) can be extracted from natural foods such as peanuts and pistachios, and in small amounts from bilberries and blueberries; REV is commonly extracted from grapes (161) and is particularly rich in grape skins and wine; REV is also found in raw cranberry juice, chocolate and products containing cocoa powder (113).

In recent years, increasing evidence has suggested that REV, as an inducer of ferroptosis can inhibit the proliferation of various tumors, such as colorectal cancer (114, 115), acute myeloid leukemia (162), lung squamous cell carcinoma (116), and canine mammary tumors (163). Regarding mechanisms of action, REV triggered an increase in ROS accumulation and lipid peroxidation in CRC cells, ultimately triggering ferroptosis. Additionally, REV enhanced ferroptosis through downregulation of channel proteins SLC7A11 and GPX4 expression (114, 115). In acute myeloid leukemia, It has been shown REV increased the expression of hsa-miR-335-5p while decreased the expression of NFS1 and GPX4; it increased ferroptosis in AML cells through the Hsa-miR-335-5p/NFS1/GPX4 pathway in a manner dependent on ROS (162). However, in lung squamous cell carcinoma, REV regulated the SLC7A11-HMMR interaction, activated ferroptosis, enhanced the cytotoxic effects on CD8^+^ T cells, and regulated the tumor immune microenvironment (116). Interestingly, NK cells pretreated with REV exhibited increased GPX4 protein expression, increasing to the sensitivity of breast cancer cells to ferroptosis and thereby promoting the antitumor activity of NK cells (163). Pilankar et al. investigated the effects of oral resveratrol and copper (R-Cu) on downregulating cancer marker features and immune checkpoints in advanced oral squamous cell carcinoma (OSCC). The study found that after two weeks of lower-dose R-Cu treatment, cellular chromatin particles (cfChPs) were cleared from the OSCC patient’s tumor microenvironment (TME), which was associated with significant downregulation of multiple biomarkers, including five immune checkpoints, thereby achieving the goal of tumor treatment. Although the number of cases included in this clinical trial was small, it paved the way for the clinical application of resveratrol in cancer treatment (159).

#### 3.3.3 Epigallocatechin-3-gallate

Epigallocatechin-3-gallate (EGCG) is a catechin, a plant-based compound, and is the most abundant and active ingredient in green tea leaves. EGCG is also naturally present in foods such as apples, blackberries, and carob flour, among many other foods (164). Previous studies have identified EGCG as a novel inducer of ferroptosis. Wang et al. reported that cell proliferation was significantly inhibited after treating NSCLC cell lines with different concentrations of EGCG. Mechanistic studies revealed that EGCG treatment led toGPX4 and SLC7A11 expression decreased, whereas the level of ACSL4 increased. These molecular changes were associated with higher levels of intracellular iron, MDA, and ROS, along with ultrastructural alterations typical of ferroptosis. EGCG also affected the ferroptosis pathway by reducing the expression of the key target tsRNA-13502 and changing the levels of important ferroptosis regulators (GPX4/SLC7A11 and ACSL4). This led to increased accumulation of iron, MDA, and ROS, ultimately triggering ferroptosis in NSCLC cells (117). Furthermore, Li et al. revealed that EGCG alleviated lung cancer progression exacerbated by obesity via the STAT1/SLC7A11 pathway and the gut microbiota via increasing the abundance of Clostridia while decreasing the abundance of Deltaproteobacteria and Epsilonproteobacteria (118).

#### 3.3.4 6-Gingerol and 6-shagaol

6-Gingerol (6G) and 6-shogaol (6-SG) are bioactive compounds derived from Zingiber officinale, and studies have demonstrated their anticancer properties against various types of cancer cells, including prostate cancer cell lines like LNCaP, PC3, and DU (119); lung cancer cells A549 (120); and endometrial cancer Ishikawa cells(159. In terms of mechanisms of action, 6G induced autophagy by significantly increasing the protein expression levels of LC3B-II and Beclin-1, significantly decreasing the protein expression levels of GPX4 and Nrf2 and increasing the ROS level in prostate cancer cells (119). 6G suppressed the growth of lung cancer cells by inhibiting USP14 expression, leading to a significant increase in the number of autophagosomes, ROS levels, and iron concentration, as well as regulating downstream autophagy-dependent ferroptosis-related proteins including nuclear receptor coactivator 4 (NCOA4), FTH1, TfR1, GPX4, and activating transcription factor 4 (ATF4) (120). 6-SG was shown to act as a ferroptosis inducer via the PI3K/AKT pathway for targeted therapy in the treatment of endometrial cancer (121). Of note, in clinical practice, persistent vomiting often makes it difficult for cancer patients to undergo standard chemotherapy, leading to treatment failure. Konmun et al. randomly assigned patients receiving moderate to highly emetogenic adjuvant chemotherapy administered alongside either 10 mg of 6G or a placebo, taken twice daily for 12 weeks. The results indicate that the 6G group had a notably higher complete response rate compared to the placebo group (P < 0.001). There was a significant difference in appetite scores (P = 0.001), which increased over time. By day 64, the FACT-G quality of life score for the 6G group was significantly higher than that for the placebo group (P < 0.001). No toxicity associated with 6G was observed. Patients receiving 6G treatment had a significantly lower incidence of grade 3 fatigue (P = 0.020). This phase II clinical trial suggests that 6G significantly improves chemotherapy-induced nausea and vomiting, overall response rate, appetite scores, and quality of life in patients, suggesting its suitability for cancer patients undergoing adjuvant chemotherapy (165).

#### 3.3.5 Juglone

Juglone (JUG) is present in the fresh ripe fruit husk, roots, leaves, and bark of walnut trees. JUG can also be found in *Carya ovata* (hickory tree), as well as in the families Proteaceae, Caesalpiniaceae, and Fabaceae. Most research has focused on Juglans nigra as the source for isolating JUG and studying the allelopathic properties of JUG due to this species producing the highest quantities of juglone (166).

JUG has been confirmed to reduce the survival rate of lung cancer A549 cells and significantly increase ferroptosis-related indicators, such as ROS, MDA, GSH, and ferrous ion levels. Mechanistically, JUG induced cell death mediated by ferroptosis, potentially accompanied by activation of HMOX1,overload of iron, inhibition of xCT, depletion of GSH and GPX4, and an increase of lipid peroxidation and accumulation of ROS, thus leading to oxidative damage and even cell death (162. Additionally, JUG induced cell death in pancreatic cancer cell line MIA-PaCa-2. However, this response was reversed by deferoxamine, suggesting that JUG induced ferroptosis, leading to the death of MIA Paca-2 cells (123). Furthermore, JUG inhibited the migration of endometrial cancer (EC) cells. Mechanistic studies have revealed that the application of walnut phenols led to accumulating Fe^2+^, increasing lipid peroxidation, depleting GSH, elevating HMOX1 expression, and degrading haem into Fe^2+^. These findings suggest that walnut phenols act as ferroptosis inducers, triggering programmed cell death in EC cells by activating oxidative stress (124). Interestingly, recently, Li et al. reported that JUG induced ferroptosis and led to inhibit the glioblastoma (GBM) progression by anchoring the Nrf2/GPX4 pathway; therefore, JUG shows potential as a new ferroptosis inducer or a treatment for anti-GBM (167).

### 3.4 Alkaloids

**Figure 7:**
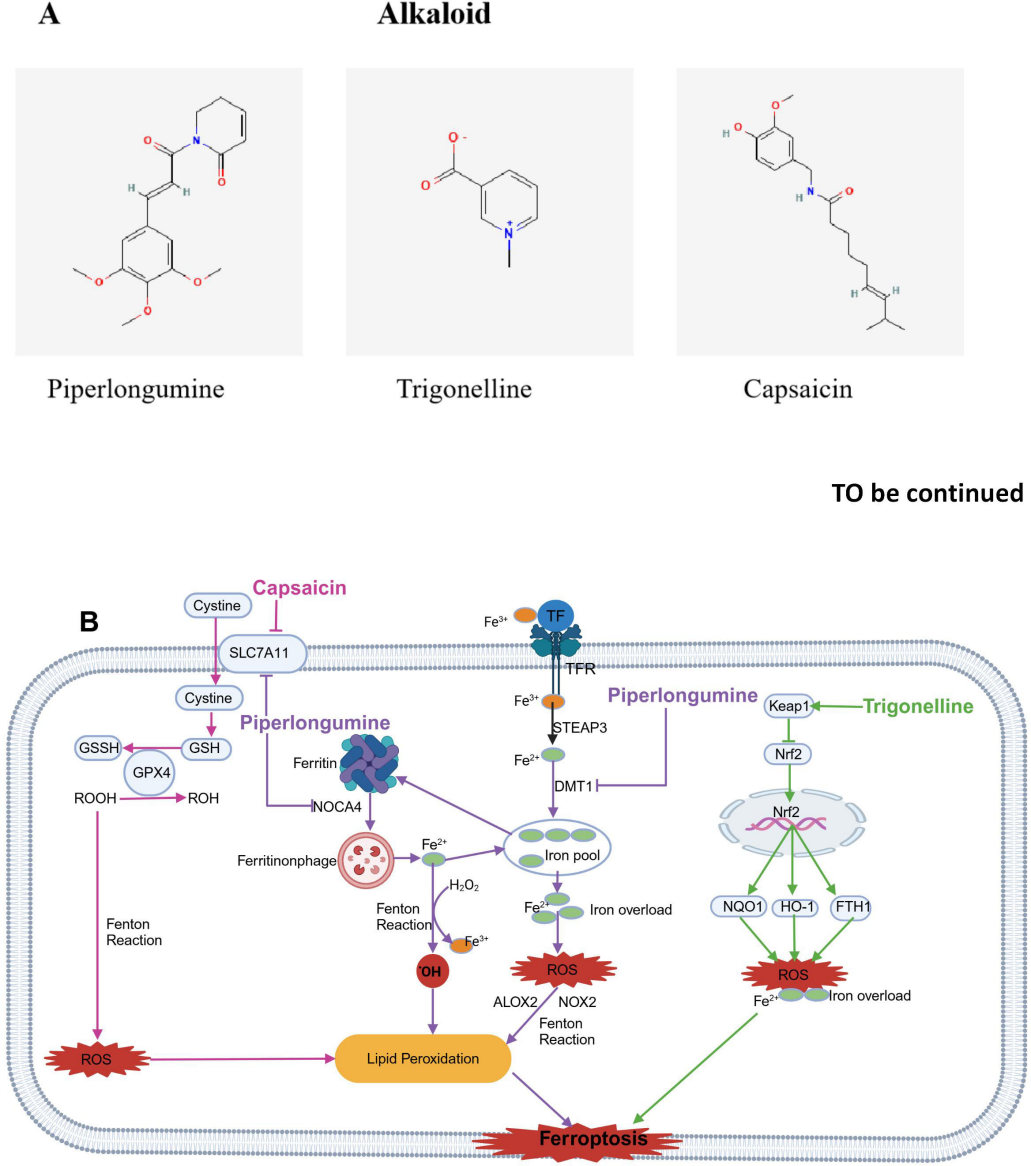
Chemical structures of food-derived alkaloids and the mechanism by which ferroptosis is induced to inhibit tumours. **(A)** Chemical structures of food-derived alkaloids. **(B)** The mechanism by which food-derived alkaloids induce ferroptosis to inhibit tumours. The map was created using biorender.

Alkaloids are organic compounds with a ring structure containing one or more basic nitrogen atoms; they exist as natural secondary metabolites in plants and animals. Alkaloids are synthesized primarily from amino acids and are present in the seeds, roots, stems, and leaves of certain higher plants, such as those in the Solanaceae, Ranunculaceae, Gentianaceae, Asparagaceae, Amaryllidaceae, and Papaveraceae families (168, 169). Increasing experimental evidence suggests that dietary alkaloids, such as piperlongumine (PL), trigonelline (TRG) and capsaicin (CAP), may serve as inducers of ferroptosis for the treatment of various tumors. The molecular structures of food-derived alkaloid compounds are illustrated in **Figure 7A**. The mechanism by which food-derived alkaloid compounds induce ferroptosis to inhibit tumors is detailed in **Figure 7B**; **Table 1**.

#### 3.4.1 Piperlongumine

Piperlongumine (PL) is an alkaloid amide derived from edible long pepper plants. The fruits of these plants are widely used in spices, pickles, preservatives, food products, beverages, alcoholic drinks, and medicines. Research indicates that PL has toxic effects on different human cancer cell lines and exhibits antitumor activity in rodents (170). Studies suggest that PL kills cancer cells by stimulating ROS production and depleting GSH in specific cancer cells without increasing ROS in normal cells (171).

Wang ZQ et al. reported that PL treatment reduced growth rate of OSCC cells in response to both dose and time. After PL treatment, lipid peroxidation and intracellular ROS accumulation increased. In addition, the expression of DMT1 was upregulated, while the expression of FTH1, SLC7A11, and GPX4 was downregulated. Furthermore, the antiproliferative activity of PL was reversed by Fer-1 and N-acetylcysteine (NAC), with corresponding decreases in LPO and ROS levels. Importantly, the combination of PL and CB-839 synergistically reduced cell viability and LPO levels, accompanied by significant GSH depletion. This evidence suggests that PL can drive ferroptosis in OSCC cells and that this effect can be enhanced by CB-839 (125). Yang Y et al. discovered that PL enhanced cancer cell ferroptosis by inhibiting TXNRD1 in colon cancer cells HCT116 cells (126). Yamaguchi et al. reported that PL caused death in a human pancreatic cancer cell line by significantly accumulating intracellular ROS and depleting GSH; Iron death inhibitors and iron chelators can block this effect, but apoptosis or necrosis inhibitors cannot, suggesting that PL may cause cell death through ferroptosis, making it a potential candidate for cancer therapy (127).

#### 3.4.2 Trigonelline

Trigonelline (TRG) is an alkaloid found in coffee beans, fenugreek seeds, various fruits and seeds. During coffee roasting, it plays an indirect role in the development of desirable flavors. Given its pharmacological benefits and low toxicity, TRG has gained increasing attention in recent years. Studies have indicated that TRG may help prevent and treat diabetes, hyperlipidemia, nervous and hormonal disorders, and cancer (172). Two studies by Dr. Roh found that, in head and neck cancer cells, TRG counteracted resistance to ferroptosis caused by RSL3 and cisplatin by inhibiting the Nrf2 pathway. Additionally, blocking GPX4 made drug-resistant cancer cells more vulnerable to ferroptosis (128, 129). Additionally, the alkaloid TRG inhibited the expression of the Nrf2 target genes such as NQO1, HO-1, as well as FTH1. Inhibiting the activation of Nrf2 can enhance the anticancer effects of Erastin and Sorafenib in hepatocellular carcinoma (HCC) cells through ferroptosiss (130). Thus, it indicated that the biological effects of TRG may be partly dependent on its ability to regulate ferroptosis.

#### 3.4.3 Capsaicin

Capsaicin (CAP) is a homovanillic acid derivative responsible for the characteristic pungency of the genus Capsicum (173). Liu et al. reported that CAP blocked the proliferation of NSCLC A549 and NCI-H23 cells and triggered ferroptosis by turning offSLC7A11/GPX4 signaling (131). Furtherly, Hacioglu C and Kar F. reported that CAP induced a redox imbalance and drove ferroptosis via the ACSL4/GPx4 pathway in glioblastoma cells U87-MG and U251 (132). In addition, research has shown that arvanil, a nonstimulatory synthetic CAP analogue, induced ferroptosis in hepatocellular carcinoma by binding to MICU1. Moreover, Arvanil increased the sensitivity of HCC cells to cisplatin *in vivo* by triggering ferroptosis (174). Recently, Chen et al. conducted a randomized controlled phase II clinical trial on the use of capsaicin to alleviate neuropathic pain in patients with head and neck cancer after treatment. The trial has not yet reached a final conclusion (175).

## 4 Progress of ferroptosis induced by food-derived compounds delivered by nanocarriers in cancer therapy

**Figure 8:**
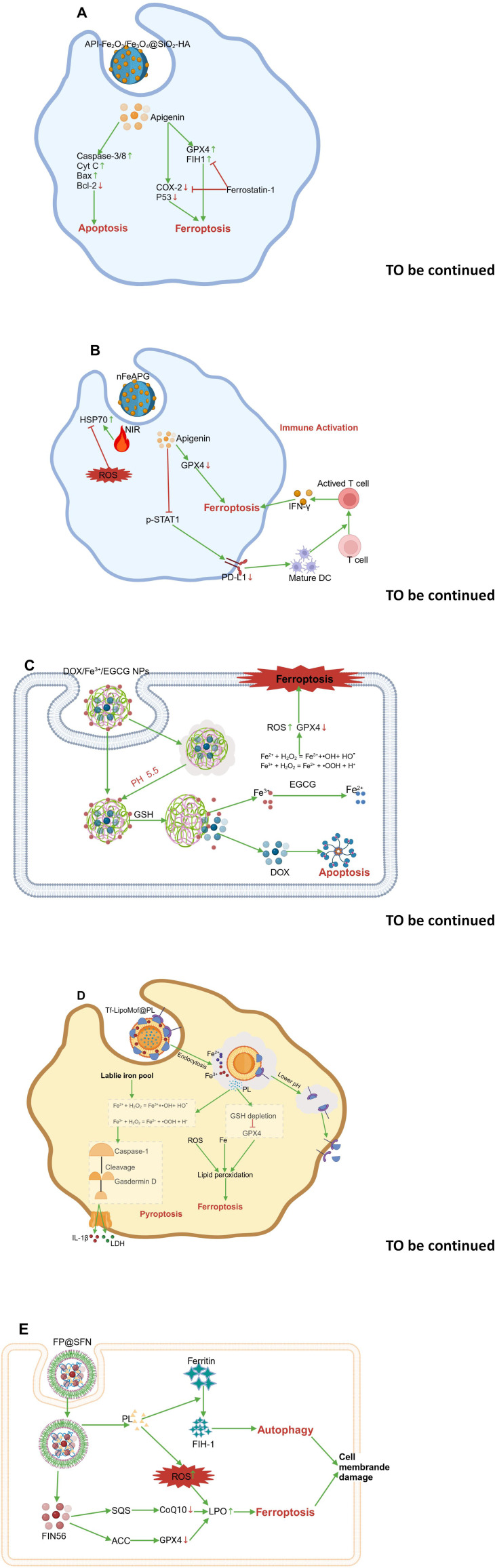
The mechanism involving inducing ferroptosis of nanocarrier-mediated delivery of food-derived compounds in cancer therapy. **(A)** API-Fe2O3/Fe3O4@SiO2-HA. **(B)** nFeAPG. **(C)** DOX/Fe3+/EGCG NPs. **(D)** Tf- LiPoMof@PL. **(E)** FP@SFN. The map was created using biorender.

A growing number of studies have shown promising outcomes in ferroptosis induced by food-derived compounds delivered by nanocarriers in cancer therapy. In this section, we review the use of nanocarriers to deliver diet-derived compounds to induce ferroptosis to improve cancer treatment (**Figures 8A–E**; **Table 3**).

**Table 3:** The nanocarrier-mediated delivery of food-derived compounds against cancer via synergestic mechanisms involving ferroptosis.

Nanosystem	Compounds	Tumor model	Synergestic mechanisms	Authors (Ref.)
Fe2O3/Fe3O4@mSiO2-HA	apigenin	A549 cells	Apoptosis and ferroptosis	Liu et al. (176)
nFeAPG	apigenin	Breast cancer	ferroptosis	Chen et al. (177)
DOX/Fe3+/EGCG(DF) nanoparticles	EGCG/DOX	LL2 and 549 cells; LL2-tumor bearing mice model	Apoptosis and ferroptosis	Mu et al. (178)
Tf-LipoMof@PL	PL	4T1 cells; 4T1 xenograft mice model	Ferroptosis and pyroptosis	Xu et al. (179)
FP@SFN	FIN56 and PL	FIN56 and PL	Ferroptosis and autophagy	Shu et al. (180)

### 4.1 Apigenin nanocomplex

Liu et al. constructed a magnetic nanomaterial system, i.e., Fe2O3/Fe3O4@mSiO2-HA. The experimental results confirmed that this anticancer drug delivery platform demonstrated strong magnetic properties, effective targeting with active hyaluronic acid, and excellent biocompatibility. Free apigenin (API) and API-Fe2O3/Fe3O4@mSiO2-HA exerted anticancer effects by promoting apoptosis in A549 cells. It was observed that API-induced expression of caspase-3, caspase-8, CytC, and Bax proteins increased, whereas the expression of Bcl-2 decreased. In addition, API-Fe2O3/Fe3O4@mSiO2-HA obviously increased the levels of ROS and cellular lipid peroxidation in A549 cells. Analyses of ferroptosis-related proteins revealed that the administration of API-Fe2O3/Fe3O4@mSiO2-HA upregulated COX2 and p53 expression and downregulated GPX4 and FTH1 expression, however, this effect was reversed by the administration of ferrostatin-1, indicating that the magnetic nanocarriers exerted drug effects through ferroptosis. Overall, magnetic nanoparticle carriers had a stronger effect than did free topotecan in promoting apoptosis and ferroptosis in tumor cells (176) (**Figure 8A**).

Additionally, Chen et al. successfully developed a novel polyphenol-metal nanostructure (nFeAPI) composed of Fe^3+^ and API. *In vitro*, the API and Fe^3+^ released from nFeAPI triggered ferroptosis, produced ROS, and decreased expression of PD-L1, demonstrating significant anticancer effects when paired with near-infrared irradiation. When nFeAPI was administered *in vivo*, it accumulated at the cancer site and was endocytosed by cancer cells. Under near-infrared irradiation, nFeAPI increased the local temperature and induced excessive ROS production through the release of API and Fe^3+^, synergistically promoting tumor cell death. The resulting tumor antigens further enhanced the maturation of dendritic cells (DCs) and the recruitment of cytotoxic T cells, thereby strengthening their anticancer effects (177) (**Figure 8B**).

### 4.2 EGCG nanoparticle

Mu and colleagues created a nanoparticle (NP) that triggered both apoptosis and ferroptosis in tumor cells. The nanoparticle, made of EGCG and Fe^3+,^ was created using a simple and eco-friendly method, enabling the delivery of both doxorubicin (DOX) and iron ions to tumor sites. DOX/Fe3+/EGCG (DF) nanoparticles have good solubility and strong long-term storage stability. Under conditions of high GHS levels and an acidic environment within tumor cells, DOX/Fe3+/EGCG (DF) nanoparticles can effectively release DOX and Fe3+. The Fe3+ released by EGCG’s chemical reduction is converted to Fe2+. The generated Fe3+/Fe2+ ions, through the Fenton reaction, convert intracellular H2O2 into hydroxyl radicals (•OH), which in turn induce ferroptosis, enhancing DOX-induced apoptosis. In both *in vitro* and *in vivo* studies, significant therapeutic effects were observed, indicating that developing a DF nanoparticle delivery system is a promising approach for fighting tumors by triggering both apoptosis and ferroptosis (178) (**Figure 8C**).

### 4.3 Piperlongumine nanodrug

The combined induction of ferroptosis and pyroptosis mechanisms offers a new approach for cancer therapy. Xu et al. proposed a dual-induction nanosystem, Tf-LipoMof@PL, made up of a metal-organic framework (MOF) containing piperlongumine (PL) and covered with a pH-sensitive lipid layer that has been modified with transferrin. PL acted as an inducer of ferroptosis, enhancing cell death and providing H_2_O_2_, which increased ROS production through the Fenton reaction in the dual-induction system. Based on the effectiveness of ferroptosis and pyroptosis combined induction, *in vivo* experiments have confirmed that dual-induction nanoparticles demonstrated ideal anti-cancer effects in a xenograft mouse model, suggesting that the combined induction of ferroptosis and pyroptosis may be an effective and promising cancer treatment approach (179) (**Figure 8D**).

Additionally, Shu et al. effectively enhanced lung cancer treatment efficacy through the synergistic induction of ferroptosis and autophagy. FP@SFN nanoparticles encapsulate FIN56 and PL in a silk protein-based nanodestroyer. PL and the novel ferroptosis inducer FIN56 are codelivered, enhancing the therapeutic effect of ferroptosis by increasing oxidative stress and connecting with the autophagy pathway. Both *in vitro* and *in vivo* studies show that FP@SFN effectively eliminates A549 cells and inhibits subcutaneous lung cancer tumors. Notably, ferroptosis and autophagy have been identified as the main mechanisms of cell death induced by the nanodystroyer, through increased oxidative stress and induction of cell membrane rupture (180) (**Figure 8E**).

To sum up, ferroptosis in targeted therapy is an exciting new area in cancer treatment, providing an innovative way to eliminate cancer cells by inducing iron-dependent lipid peroxidation. Current research on food-derived compounds as inducers of ferroptosis to suppress tumors enhances our comprehension and application of this process within the realm of precision oncology. In a clinical setting, assessing potential biomarkers for ferroptosis is of significant importance for monitoring the pathological mechanisms in patients. While Certain unique biochemical traits, genetic modifications, and changes in cell morphology can distinguish ferroptosis from other forms of regulated cell death (RCDs). Effectively monitoring ferroptosis in living organisms or identifying cells that are sensitive to it holds significant potential. Fortunately, a review article by Chen and his team provides a comprehensive overview of ferroptosis biomarkers (181). The article points out that the buildup of lipid peroxides is a key event in ferroptosis. Specific markers for ferroptosis include oxidized polyunsaturated fatty acid phospholipids and their breakdown products, as well as oxidative compounds such as 4-HNE, 8-hydroxy-2′-deoxyguanosine (8-OHdG), and MDA. However, the difficulty arises because these substances can also be produced under normal physiological conditions. Therefore, it is essential to identify the point at which ferroptosis begins to occur in living organisms (181). Overoxidized peroxiredoxin 3 (PRDX3) has become a specific marker for ferroptosis, distinguishing it from other RCDs (181). Additionally, fluorescent probes such as QP1TF can continuously assess the distribution and abundance of GPX4, and GSH-reversible probes can monitor the process of intracellular lipid peroxidation as ferroptosis biomarkers (181). Quantitative PCR (qPCR) detection of gene expression markers during ferroptosis, such as CHAC1, PTGS2, SLC7A11, ACSL4, and RGS4, can also serve as ferroptosis biomarkers (181). Therefore, through the rational selection and combined use of various biomarkers and detection methods, it is possible to better monitor the pathological mechanisms in patients and assess the tumor’s sensitivity to and efficacy of treatment.

## 5 Conclusion and future prospects

Ferroptosis is a form of cell death that depends on iron and is mainly triggered by excessive lipid peroxidation, a process driven by harmful ROS (reactive oxygen species) formed through iron. Recently, in-depth research on the mechanisms and targets of ferroptosis has led to the development of many ferroptosis inducers, particularly food-derived bioactive compounds, have been identified and developed. These compounds have been validated in various tumor models, showing that they inhibit tumor progression by inducing ferroptosis. In addition, food-derived bioactive compounds provide benefits like high safety and easyavailability. Therefore, ferroptosis inducers derived from food bioactive compounds hold promise as potential options for cancer treatment through dietary supplementation or other approaches.

In this article, we introduced the molecular mechanisms of ferroptosis and the application of various food-derived compounds as ferroptosis inducers in cancer therapy. A comprehensive and systematic summary of food-derived ferroptosis inducers was provided, offering insights for the design and optimization of new ferroptosis-inducing drugs. Research progress on bioactive food compounds in nanomaterials through the induction of ferroptosis and synergistic mechanisms involving ferroptosis was also summarized, providing a reference for the development and clinical application of ferroptosis inducers in emerging areas.

Despite significant progress in the use of dietary compounds to induce ferroptosis in cancer therapy, there are still notable limitations. (1) Most of the dietary compounds exhibit poor absorption, uneven distribution, abnormal metabolism, and specific excretion characteristics, with low water solubility, necessitating further chemical modifications or encapsulation in targeted delivery systems. Although several studies have demonstrated the effectiveness of the nanotechnology-based targeted delivery of dietary compounds, more studies are required to confirm the findings. (2) The functional targets of the dietary compounds as ferroptosis inducers remain largely unclear. Further research may help identify new ferroptosis regulatory mechanisms through chemobiological approaches. (3) Before conducting randomized clinical trials, it is essential to use nanotechnology to enhance targeted drug delivery, control drug release, and increase drug solubility and bioavailability and to conduct necessary pharmacokinetic, pharmacodynamic, and toxicological studies.

The low bioavailability of food-derived compounds (such as curcumin and resveratrol) is a major bottleneck in their clinical translation. It is proposed that this bottleneck can be overcome through several approaches, maximizing their potential in disease treatment. (1) Develop new carrier systems. In the future, more efficient and stable new carrier systems can be developed, such as nanoparticle carriers based on biodegradable materials or smart-responsive carriers, to further enhance the bioavailability of food-derived compounds. These new carrier systems can intelligently release drugs according to changes in the physiological environment in the body, increasing the concentration of the drug in the target tissue (182). (2) Explore combination therapy strategies. By combining with other drugs, the bioavailability and therapeutic effects of curcumin and resveratrol can be synergistically enhanced. For example, using drugs that inhibit metabolic enzyme activity can reduce the metabolism of curcumin and resveratrol in the body, extending their duration of action (183–185). (3) Develop personalized treatment plans. Due to significant physiological differences between individuals, future treatments can be tailored to the patient’s unique characteristics, such as genetic background and gut microbiome composition, to enhance the effectiveness of curcumin and resveratrol. For example, through genetic testing, patients who metabolize curcumin and resveratrol more slowly can be identified and prescribed higher doses of the medication (186).

In the future, after addressing issues like the bioavailability of dietary compounds through the methods mentioned above, enhancing the synergistic treatment of tumors between dietary compounds and traditional therapies will also be a key focus in the research of dietary compounds. Excitingly, preclinical trials have shown that dietary compounds have made significant progress in counteracting chemotherapy drug resistance and improving the effectiveness of both radiotherapy and chemotherapy for tumors has been made. For example, curcumin has been demonstrated to overcome the resistance of B cells in chronic lymphocytic leukemia (CLL) to chemotherapy drugs. When curcumin is used in combination with chemotherapy drugs (such as capecitabine), it can significantly enhance the pro-apoptotic effects of chemotherapy and inhibit NF-κB activity. Additionally, curcumin can regulate the sensitivity of colon cancer cells to radiotherapy, enhancing the effects of radiotherapy by inhibiting the activation of the NF-κB pathway (187–189).

## Glossary

GPX4: glutathione peroxidase 4
TF: transferrin
TFR1: transferrin receptor 1
STEAP3: six-transmembrane epithelial antigen of the prostate 3
ALOX: arachidonate lipoxygenases
HMOX1: heme oxygenase 1
HNE: 4-hydroxynonenal
MDA: malondialdehyde
LOOHs: lipid hydroperoxide
AA: arachidonic acid
AdA: adrenic acid
ACSL4: long-chain acyl-CoA synthetase 4
PE: phosphatidylethanolamine
H_2_O_2_: hydrogen peroxide
Fe^2+^: ferrous iron
Fe^3+^: ferric iron
NOX: NADPH oxidase
PUFAs: polyunsaturated fatty acids
PLOOH: phospholipid hydroperoxides
LOX: lipoxygenase
GSH: glutathione
System x_c_^−^: cysteine/glutamate antiporter
xCT/SLC7A11: solute carrier family 7 member 11
FSP1: ferroptosis suppressor protein 1
CoQ1: coenzyme Q10
NAD(P)H: nicotinamide adenine dinucleotide phosphate
CoQH2: Coenzyme Q10
BH4: dihydrofolate to tetrahydrofolate
Nrf2: nuclear factor erythroid 2-related factor 2
Keap1: Kelch-like ECH-associated protein 1
ARE: antioxidant response elements
GCL: glutamate cysteine ligase
GSS: glutathione synthetase
HO-1: heme oxygenase-1
USP7: ubiquitin-specific protease 7
SAT1: spermidine/spermine N1-acetyltransferase 1
IPP: isopentenyl pyrophosphate
MVA pathway: mevalonate pathway
GCLC: glutamate-cysteine ligase catalytic subunit
α-KG: α-ketoglutarate
VDAC2/3: voltage-dependent anion channels2/3
ETC: electron transfer chain
ROS: reactive oxygen species
STAT3: transcription 3
Fer-1: ferroptosis inhibitor ferrostatin-1
GRh3: Ginsenoside Rh3
GRh4: Ginsenoside Rh4
GRg5: Ginsenoside Rg5
GRK1: Ginsenoside RK1
GCK: Ginsenoside CK
NCOA4: nuclear receptor coactivator 4
NR3C1: nuclear receptor subfamily 3 group C member 1
FOXO1: forkhead box protein O1
PLOO^•^: phospholipid peroxyl radicals
AMPK: activated protein kinase
PGC1α: peroxisome proliferators-activated receptor γ
TRIB3: tribbles protein kinase 3
p-DRP1: phospho dynamin-related protein 1
GSK3β: glycogen synthase kinase-3 beta
NQO1: non-Q-dependent Reductase
PDK1: pyruvate dehydrogenase kinase 1
LDH: lactate dehydrogenase
TCA cycle: tricarboxylic acid cycle
TCF4: T cell factor 4
ALOXE3: arachidonate lipoxygenase 3
HIC1: hypermethylated in cancer 1
LAMP-1: lysosomal associated membrane protein 1
SOD4: superoxide dismutase 4
GDF15: growth differentiation factor 15
LIP: labile iron pool
DRP1: dynamin-related protein 1
Camk2: calcium/calmodulin-dependent protein kinase II
NQO1: non-Q-dependent Reductase
TCA cycle: tricarboxylic acid cycle;GPX4, glutathione peroxidase 4
^•^OH: hydroxyl radicals
O_2_^•–^: superoxide radicals
GSSH: oxidized glutathione
DMT1: divalent metal transporter 1
LPO: lipid peroxidation
STAT1: transcription 1
COX-2: Cyclooxygenase-2
FTH1: ferritin heavy chain 1
API: APG, apigenin
Cyt c: Cytochrome C
Bax: BCL2-Associated X Protein
Bcl-2: B-cell lymphoma-2
HSP70: heat shock protein 70
NIR: NearInfrared
INF-γ: interferon-γ
PD-L1: programmed cell death ligand 1
DC: dendritic cells
DOX: doxorubicin
EGCG: epigallocatechin-3-gallate
NPs: nanoparticles
PL: piperlongumine
LDH: lactate dehydrogenase
SQS: squalene synthase
ACC: acetyl-CoA carboxylase.

## References

[1] Bray F Laversanne M Sung H Ferlay J Siegel RL Soerjomataram I. Global cancer statistics 2022: GLOBOCAN estimates of incidence and mortality worldwide for 36 cancers in 185 countries. CA Cancer J Clin. (2024) 74:229–63. doi: 10.3322/caac.21834 38572751

[2] Zeng H Chen W Zheng R Zhang S Ji JS Zou X. Changing cancer survival in China during 2003-15: a pooled analysis of 17 population-based cancer registries. Lancet Glob Health. (2018) 6:e555–67. doi: 10.1016/S2214-109X(18)30127-X 29653628

[3] Maomao C He L Dianqin S Siyi H Xinxin Y Fan Y. Current cancer burden in China: epidemiology, etiology, and prevention. Cancer Biol Med. (2022) 19:1121–38. doi: 10.20892/j.issn.2095-3941.2022.0231 PMC9425189 36069534

[4] Ferlay J Colombet M Soerjomataram I Parkin DM Piñeros M Znaor A. Cancer statistics for the year 2020: An overview. Int J Cancer. (2021). doi: 10.1002/ijc.33588 33818764

[5] Torre LA Bray F Siegel RL Ferlay J Lortet-Tieulent J Jemal A. Global cancer statistics, 2012. CA Cancer J Clin. (2015) 65:87–108. doi: 10.3322/caac.21262 25651787

[6] Xi Y Xu P. Global colorectal cancer burden in 2020 and projections to 2040. Transl Oncol. (2021) 14:101174. doi: 10.1016/j.tranon.2021.101174 34243011 PMC8273208

[7] Didkowska J Wojciechowska U Mańczuk M Łobaszewski J. Lung cancer epidemiology: contemporary and future challenges worldwide. Ann Transl Med. (2016) 4:150. doi: 10.21037/atm.2016.03.11 27195268 PMC4860480

[8] McCormack VA Boffetta P. Today’s lifestyles, tomorrow’s cancers: trends in lifestyle risk factors for cancer in low- and middle-income countries. Ann Oncol. (2011) 22:2349–57. doi: 10.1093/annonc/mdq763 21378201

[9] Zhang P Jing QG Zhang YH Jin WD Wang Y Du J. Immunotherapies of acute myeloid leukemia: rationale, clinical evidence and perspective. BioMed Pharmacother. (2024) 171:116132. doi: 10.1016/j.biopha.2024.116132 38198961

[10] Dixon SJ Lemberg KM Lamprecht MR Skouta R Zaitsev EM Gleason CE. Ferroptosis: an iron-dependent form of nonapoptotic cell death. Cell. (2012) 149:1060–72. doi: 10.1016/j.cell.2012.03.042 PMC3367386 22632970

[11] Chen X Li J Kang R Klionsky DJ Tang D. Ferroptosis: machinery and regulation. Autophagy. (2021) 17:2054–81. doi: 10.1080/15548627.2020.1810918 PMC8496712 32804006

[12] Su Y Zhao B Zhou L Zhang Z Shen Y Lv H. Ferroptosis, a novel pharmacological mechanism of anti-cancer drugs. Cancer Lett. (2020) 483:127–36. doi: 10.1016/j.canlet.2020.02.015 32067993

[13] Zhang R Chen J Wang S Zhang W Zheng Q Cai R. Ferroptosis in cancer progression. Cells. (2023) 12:1820. doi: 10.3390/cells12141820 37508485 PMC10378139

[14] Yan H Talty R Aladelokun O Bosenberg M Johnson CH. Ferroptosis in colorectal cancer: a future target. Br J Cancer. (2023) 128:1439–51. doi: 10.1038/s41416-023-02149-6 PMC10070248 36703079

[15] Jiang L Kon N Li T Wang SJ Su T Hibshoosh H. Ferroptosis as a p53-mediated activity during tumor suppression. Nature. (2015) 520:57–62. doi: 10.1038/nature14344 25799988 PMC4455927

[16] Zhang Y Shi J Liu X Feng L Gong Z Koppula P. BAP1 links metabolic regulation of ferroptosis to tumor suppression. Nat Cell Biol. (2018) 20:1181–92. doi: 10.1038/s41556-018-0178-0 PMC6170713 30202049

[17] Gao M Yi J Zhu J Minikes AM Monian P Thompson CB. Role of mitochondria in ferroptosis. Mol Cell. (2019) 73:354–363.e3. doi: 10.1016/j.molcel.2018.10.042 30581146 PMC6338496

[18] Mou Y Wang J Wu J He D Zhang C Duan C. Ferroptosis, a new form of cell death: opportunities and challenges in cancer. J Hematol Oncol. (2019) 12:34. doi: 10.1186/s13045-019-0720-y 30925886 PMC6441206

[19] Jiang X Stockwell BR Conrad M. Ferroptosis: mechanisms, biology and role in disease. Nat Rev Mol Cell Biol. (2021) 22:266–82. doi: 10.1038/s41580-020-00324-8 PMC8142022 33495651

[20] Guo J Xu B Han Q Zhou H Xia Y Gong C. Ferroptosis: A novel anti-tumor action for cisplatin. Cancer Res Treat. (2018) 50:445–60. doi: 10.4143/crt.2016.572 PMC5912137 28494534

[21] Lachaier E Louandre C Godin C Saidak Z Baert M Diouf M. Sorafenib induces ferroptosis in human cancer cell lines originating from different solid tumors. Anticancer Res. (2014) 34:6417–22. 25368241

[22] Ma S Henson ES Chen Y Gibson SB. Ferroptosis is induced following siramesine and lapatinib treatment of breast cancer cells. Cell Death Dis. (2016) 7:e2307. doi: 10.1038/cddis.2016.208 27441659 PMC4973350

[23] Xie Y Hou W Song X Yu Y Huang J Sun X. Ferroptosis: process and function. Cell Death Differ. (2016) 23:369–79. doi: 10.1038/cdd.2015.158 PMC5072448 26794443

[24] Kang H Han M Xue J Baek Y Chang J Hu S. Renal clearable nanochelators for iron overload therapy. Nat Commun. (2019) 10:5134. doi: 10.1038/s41467-019-13143-z 31723130 PMC6853917

[25] Wu Z Zhong M Liu Y Xiong Y Gao Z Ma J. Application of natural products for inducing ferroptosis in tumor cells. Biotechnol Appl Biochem. (2022) 69:190–7. doi: 10.1002/bab.2096 33393679

[26] Hassannia B Wiernicki B Ingold I Qu F Van Herck S Tyurina YY. Nano-targeted induction of dual ferroptotic mechanisms eradicates high-risk neuroblastoma. J Clin Invest. (2018) 128:3341–55. doi: 10.1172/JCI99032 PMC6063467 29939160

[27] Tang D Chen X Kang R Kroemer G. Ferroptosis: molecular mechanisms and health implications. Cell Res. (2021) 31:107–25. doi: 10.1038/s41422-020-00441-1 PMC8026611 33268902

[28] Galy B Conrad M Muckenthaler M. Mechanisms controlling cellular and systemic iron homeostasis. Nat Rev Mol Cell Biol. (2024) 25:133–55. doi: 10.1038/s41580-023-00648-1 37783783

[29] Stoyanovsky DA Tyurina YY Shrivastava I Bahar I Tyurin VA Protchenko O. Iron catalysis of lipid peroxidation in ferroptosis: Regulated enzymatic or random free radical reaction. Free Radic Biol Med. (2019) 133:153–61. doi: 10.1016/j.freeradbiomed.2018.09.008 PMC6555767 30217775

[30] Mancias JD Wang X Gygi SP Harper JW Kimmelman AC. Quantitative proteomics identifies NCOA4 as the cargo receptor mediating ferritinophagy. Nature. (2014) 509:105–9. doi: 10.1038/nature13148 PMC4180099 24695223

[31] Campbell NK Fitzgerald HK Dunne A. Regulation of inflammation by the antioxidant haem oxygenase 1. Nat Rev Immunol. (2021) 21:411–25. doi: 10.1038/s41577-020-00491-x 33514947

[32] Nemeth E Tuttle MS Powelson J Vaughn MB Donovan A Ward DM. Hepcidin regulates cellular iron efflux by binding to ferroportin and inducing its internalization. Science. (2004) 306:2090–3. doi: 10.1126/science.1104742 15514116

[33] Yu Y Jiang L Wang H Shen Z Cheng Q Zhang P. Hepatic transferrin plays a role in systemic iron homeostasis and liver ferroptosis. Blood. (2020) 136:726–39. doi: 10.1182/blood.2019002907 PMC7414596 32374849

[34] Hong X Roh W Sullivan RJ Wong KHK Wittner BS Guo H. The lipogenic regulator SREBP2 induces transferrin in circulating melanoma cells and suppresses ferroptosis. Cancer Discov. (2021) 11:678–95. doi: 10.1158/2159-8290.CD-19-1500 PMC7933049 33203734

[35] Feng H Stockwell BR. Unsolved mysteries: How does lipid peroxidation cause ferroptosis. PloS Biol. (2018) 16:e2006203. doi: 10.1371/journal.pbio.2006203 29795546 PMC5991413

[36] Doll S Proneth B Tyurina YY Panzilius E Kobayashi S Ingold I. ACSL4 dictates ferroptosis sensitivity by shaping cellular lipid composition. Nat Chem Biol. (2017) 13:91–8. doi: 10.1038/nchembio.2239 PMC5610546 27842070

[37] Reed A Ichu TA Milosevich N Melillo B Schafroth MA Otsuka Y. LPCAT3 inhibitors remodel the polyunsaturated phospholipid content of human cells and protect from ferroptosis. ACS Chem Biol. (2022) 17:1607–18. doi: 10.1021/acschembio.2c00317 35658397

[38] Fang X Ardehali H Min J Wang F. The molecular and metabolic landscape of iron and ferroptosis in cardiovascular disease. Nat Rev Cardiol. (2023) 20:7–23. doi: 10.1038/s41569-022-00735-4 35788564 PMC9252571

[39] Huang Y Sarkhel S Roy A Mohan A. Interrelationship of lipid aldehydes (MDA, 4-HNE, and 4-ONE) mediated protein oxidation in muscle foods. Crit Rev Food Sci Nutr. (2024) 64:11809–25. doi: 10.1080/10408398.2023.2245029 37589270

[40] Zou Y Li H Graham ET Deik AA Eaton JK Wang W. Cytochrome P450 oxidoreductase contributes to phospholipid peroxidation in ferroptosis. Nat Chem Biol. (2020) 16:302–9. doi: 10.1038/s41589-020-0472-6 PMC7353921 32080622

[41] Yant LJ Ran Q Rao L Van Remmen H Shibatani T Belter JG. The selenoprotein GPX4 is essential for mouse development and protects from radiation and oxidative damage insults. Free Radic Biol Med. (2003) 34:496–502. doi: 10.1016/s0891-5849(02)01360-6 12566075

[42] Mao C Liu X Zhang Y Lei G Yan Y Lee H. DHODH-mediated ferroptosis defence is a targetable vulnerability in cancer. Nature. (2021) 593:586–90. doi: 10.1038/s41586-021-03539-7 PMC8895686 33981038

[43] Ursini F Maiorino M. Lipid peroxidation and ferroptosis: The role of GSH and GPx4. Free Radic Biol Med. (2020) 152:175–85. doi: 10.1016/j.freeradbiomed.2020.02.027 32165281

[44] Koppula P Zhuang L Gan B. Cystine transporter SLC7A11/xCT in cancer: ferroptosis, nutrient dependency, and cancer therapy. Protein Cell. (2021) 12:599–620. doi: 10.1007/s13238-020-00789-5 33000412 PMC8310547

[45] Yang WS SriRamaratnam R Welsch ME Shimada K Skouta R Viswanathan VS. Regulation of ferroptotic cancer cell death by GPX4. Cell. (2014) 156:317–31. doi: 10.1016/j.cell.2013.12.010 PMC4076414 24439385

[46] Yan R Xie E Li Y Li J Zhang Y Chi X. The structure of erastin-bound xCT-4F2hc complex reveals molecular mechanisms underlying erastin-induced ferroptosis. Cell Res. (2022) 32:687–90. doi: 10.1038/s41422-022-00642-w PMC9253326 35352032

[47] Doll S Freitas FP Shah R Aldrovandi M da Silva MC Ingold I. FSP1 is a glutathione-independent ferroptosis suppressor. Nature. (2019) 575:693–8. doi: 10.1038/s41586-019-1707-0 31634899

[48] Frei B Kim MC Ames BN. Ubiquinol-10 is an effective lipid-soluble antioxidant at physiological concentrations. Proc Natl Acad Sci U S A. (1990) 87:4879–83. doi: 10.1073/pnas.87.12.4879 PMC54222 2352956

[49] Bersuker K Hendricks JM Li Z Magtanong L Ford B Tang PH. The CoQ oxidoreductase FSP1 acts parallel to GPX4 to inhibit ferroptosis. Nature. (2019) 575:688–92. doi: 10.1038/s41586-019-1705-2 PMC6883167 31634900

[50] Song Y Qu Y Mao C Zhang R Jiang D Sun X. Post-translational modifications of Keap1: the state of the art. Front Cell Dev Biol. (2024) 11:1332049. doi: 10.3389/fcell.2023.1332049 38259518 PMC10801156

[51] Gong Z Xue L Li H Fan S van Hasselt CA Li D. Targeting Nrf2 to treat thyroid cancer. BioMed Pharmacother. (2024) 173:116324. doi: 10.1016/j.biopha.2024.116324 38422655

[52] Fan Z Wirth AK Chen D Wruck CJ Rauh M Buchfelder M. Nrf2-Keap1 pathway promotes cell proliferation and diminishes ferroptosis. Oncogenesis. (2017) 6:e371. doi: 10.1038/oncsis.2017.65 28805788 PMC5608917

[53] Chan JY Kwong M. Impaired expression of glutathione synthetic enzyme genes in mice with targeted deletion of the Nrf2 basic-leucine zipper protein. Biochim Biophys Acta. (2000) 1517:19–26. doi: 10.1016/s0167-4781(00)00238-4 11118612

[54] Li B Nasser MI Masood M Adlat S Huang Y Yang B. Efficiency of Traditional Chinese medicine targeting the Nrf2/HO-1 signaling pathway. BioMed Pharmacother. (2020) 126:110074. doi: 10.1016/j.biopha.2020.110074 32163746

[55] Zhang J Zhang L Yao G Zhao H Wu S. NRF2 is essential for iron-overload stimulated osteoclast differentiation through regulation of redox and iron homeostasis. Cell Biol Toxicol. (2023) 39:3305–21. doi: 10.1007/s10565-023-09834-5 37855941

[56] Wang Y Yang L Zhang X Cui W Liu Y Sun QR. Epigenetic regulation of ferroptosis by H2B monoubiquitination and p53. EMBO Rep. (2019) 20:e47563. doi: 10.15252/embr.201847563 31267712 PMC6607012

[57] Thomas T Thomas TJ. Polyamine metabolism and cancer. J Cell Mol Med. (2003) 7:113–26. doi: 10.1111/j.1582-4934.2003.tb00210.x PMC6740079 12927050

[58] Mandal S Mandal A Park MH. Depletion of the polyamines spermidine and spermine by overexpression of spermidine/spermine N¹-acetyltransferase 1 (SAT1) leads to mitochondria-mediated apoptosis in mammalian cells. Biochem J. (2015) 468:435–47. doi: 10.1042/BJ20150168 PMC4550555 25849284

[59] Ou Y Wang SJ Li D Chu B Gu W. Activation of SAT1 engages polyamine metabolism with p53-mediated ferroptotic responses. Proc Natl Acad Sci U S A. (2016) 113:E6806–12. doi: 10.1073/pnas.1607152113 PMC5098629 27698118

[60] Lee J Roh JL. Targeting GPX4 in human cancer: Implications of ferroptosis induction for tackling cancer resilience. Cancer Lett. (2023) 559:216119. doi: 10.1016/j.canlet.2023.216119 36893895

[61] Liao P Hemmerlin A Bach TJ Chye ML. The potential of the mevalonate pathway for enhanced isoprenoid production. Biotechnol Adv. (2016) 34:697–713. doi: 10.1016/j.bioteChadv.2016.03.005 26995109

[62] Zheng J Conrad M. The metabolic underpinnings of ferroptosis. Cell Metab. (2020) 32:920–37. doi: 10.1016/j.cmet.2020.10.011 33217331

[63] McGivan JD Bungard CI. The transport of glutamine into mammalian cells. Front Biosci. (2007) 12:874–82. doi: 10.2741/2109 17127344

[64] Kang YP Mockabee-Macias A Jiang C Falzone A Prieto-Farigua N Stone E. Non-canonical glutamate-cysteine ligase activity protects against ferroptosis. Cell Metab. (2021) 33:174–189.e7. doi: 10.1016/j.cmet.2020.12.007 33357455 PMC7839835

[65] Gao M Jiang X. To eat or not to eat-the metabolic flavor of ferroptosis. Curr Opin Cell Biol. (2018) 51:58–64. doi: 10.1016/j.ceb.2017.11.001 29175614 PMC5949249

[66] Baker MA Lane DJ Ly JD De Pinto V Lawen A. VDAC1 is a transplasma membrane NADH-ferricyanide reductase. J Biol Chem. (2004) 279:4811–9. doi: 10.1074/jbc.M311020200 14573604

[67] Graham BH Craigen WJ. Genetic approaches to analyzing mitochondrial outer membrane permeability. Curr Top Dev Biol. (2004) 59:87–118. doi: 10.1016/S0070-2153(04)59004-X 14975248

[68] Zhao Y Li Y Zhang R Wang F Wang T Jiao Y. The role of erastin in ferroptosis and its prospects in cancer therapy. Onco Targets Ther. (2020) 13:5429–41. doi: 10.2147/OTT.S254995 PMC7295539 32606760

[69] Heslop KA Milesi V Maldonado EN. VDAC modulation of cancer metabolism: advances and therapeutic challenges. Front Physiol. (2021) 12:742839. doi: 10.3389/fphys.2021.742839 34658929 PMC8511398

[70] Yagoda N von Rechenberg M Zaganjor E Bauer AJ Yang WS Fridman DJ. RAS-RAF-MEK-dependent oxidative cell death involving voltage-dependent anion channels. Nature. (2007) 447:864–8. doi: 10.1038/nature05859 PMC3047570 17568748

[71] Ahmad R Khan MA Srivastava AN Gupta A Srivastava A Jafri TR. Anticancer potential of dietary natural products: A comprehensive review. Anticancer Agents Med Chem. (2020) 20:122–236. doi: 10.2174/1871520619666191015103712 31749433

[72] Mehta J Rayalam S Wang X. Cytoprotective effects of natural compounds against oxidative stress. Antioxidants (Basel). (2018) 7:147. doi: 10.3390/antiox7100147 30347819 PMC6210295

[73] Diniz do Nascimento L Moraes AAB Costa KSD Pereira Galúcio JM Taube PS Costa CML. Bioactive natural compounds and antioxidant activity of essential oils from spice plants: new findings and potential applications. Biomolecules. (2020) 10:988. doi: 10.3390/biom10070988 32630297 PMC7407208

[74] Wu Y Pi D Zhou S Yi Z Dong Y Wang W. Ginsenoside Rh3 induces pyroptosis and ferroptosis through the Stat3/p53/NRF2 axis in colorectal cancer cells. Acta Biochim Biophys Sin (Shanghai). (2023) 55:587–600. doi: 10.3724/abbs.2023068 37092860 PMC10195137

[75] Wu Y Pi D Chen Y Zuo Q Zhou S Ouyang M. Ginsenoside rh4 inhibits colorectal cancer cell proliferation by inducing ferroptosis via autophagy activation. Evid Based Complement Alternat Med. (2022) 2022:6177553. doi: 10.1155/2022/6177553 35677385 PMC9168088

[76] Ying Q Lou J Zheng D. Ginsenoside Rh4 inhibits the Malignant progression of multiple myeloma and induces ferroptosis by regulating SIRT2. Clin Exp Pharmacol Physiol. (2023) 50:757–65. doi: 10.1111/1440-1681.13805 37452691

[77] Zhao H Ding R Han J. Ginsenoside rh4 facilitates the sensitivity of renal cell carcinoma to ferroptosis via the NRF2 pathway. Arch Esp Urol. (2024) 77:119–28. doi: 10.56434/j.arch.esp.urol.20247702.16 38583003

[78] Zhang G Hu J Li A Zhang H Guo Z Li X. Ginsenoside Rg5 inhibits glioblastoma by activating ferroptosis via NR3C1/HSPB1/NCOA4. Phytomedicine. (2024) 129:155631. doi: 10.1016/j.phymed.2024.155631 38640858

[79] Chen J Wang Z Fu J Cai Y Cheng H Cui X. Ginsenoside compound K induces ferroptosis via the FOXO pathway in liver cancer cells. BMC Complement Med Ther. (2024) 24:174. doi: 10.1186/s12906-024-04471-9 38664638 PMC11044296

[80] Jiang Y Yu Y Pan Z Wang Z Sun M. Ginsenoside RK1 induces ferroptosis in hepatocellular carcinoma cells through an FSP1-dependent pathway. Pharm (Basel). (2024) 17:871. doi: 10.3390/ph17070871 PMC11279434 39065721

[81] Wang X Chen J Tie H Tian W Zhao Y Qin L. Eriodictyol regulated ferroptosis, mitochondrial dysfunction, and cell viability via Nrf2/HO-1/NQO1 signaling pathway in ovarian cancer cells. J Biochem Mol Toxicol. (2023) 37:e23368. doi: 10.1002/jbt.23368 37020356

[82] Li YZ Deng J Zhang XD Li DY Su LX Li S. Naringenin enhances the efficacy of ferroptosis inducers by attenuating aerobic glycolysis by activating the AMPK-PGC1α signalling axis in liver cancer. Heliyon. (2024) 10:e32288. doi: 10.1016/j.heliyon.2024.e32288 38912485 PMC11190665

[83] Chen L Lin W Zhang H Geng S Le Z Wan F. TRIB3 promotes Malignancy of head and neck squamous cell carcinoma via inhibiting ferroptosis. Cell Death Dis. (2024) 15:178. doi: 10.1038/s41419-024-06472-5 38429254 PMC10907716

[84] Lv Y Liu Z Deng L Xia S Mu Q Xiao B. Hesperetin promotes bladder cancer cells death via the PI3K/AKT pathway by network pharmacology and molecular docking. Sci Rep. (2024) 14:1009. doi: 10.1038/s41598-023-50476-8 38200039 PMC10781778

[85] Adham AN Adham AN Abdelfatah S Naqishbandi AM Mahmoud N Efferth T. Cytotoxicity of apigenin toward multiple myeloma cell lines and suppression of iNOS and COX-2 expression in STAT1-transfected HEK293 cells. Phytomedicine. (2021) 80:153371. doi: 10.1016/j.phymed.2020.153371 33070080

[86] Wang ZX Ma J Li XY Wu Y Shi H Chen Y. Quercetin induces p53-independent cancer cell death through lysosome activation by the transcription factor EB and Reactive Oxygen Species-dependent ferroptosis. Br J Pharmacol. (2021) 178:1133–48. doi: 10.1111/bph.15350 33347603

[87] Ding L Dang S Sun M Zhou D Sun Y Li E. Quercetin induces ferroptosis in gastric cancer cells by targeting SLC1A5 and regulating the p-Camk2/p-DRP1 and NRF2/GPX4 Axes. Free Radic Biol Med. (2024) 213:150–63. doi: 10.1016/j.freeradbiomed.2024.01.002 38190923

[88] Qin X Zhang J Wang B Xu G Yang X Zou Z. Ferritinophagy is involved in the zinc oxide nanoparticles-induced ferroptosis of vascular endothelial cells. Autophagy. (2021) 17:4266–85. doi: 10.1080/15548627.2021.1911016 PMC8726675 33843441

[89] An S Hu M. Quercetin promotes TFEB nuclear translocation and activates lysosomal degradation of ferritin to induce ferroptosis in breast cancer cells. Comput Intell Neurosci. (2022) 2022:5299218. doi: 10.1155/2022/5299218 35898781 PMC9313917

[90] Tian W Wan X Tian L Wu Y Cui X Yi J. New molecular insights into ferroptosis in lung adenocarcinoma progression and pharmacological compounds for targeted therapy. J Gene Med. (2024) 26:e3579. doi: 10.1002/jgm.3579 37581210

[91] Feng S Zhou Y Huang H Lin Y Zeng Y Han S. Nobiletin induces ferroptosis in human skin melanoma cells through the GSK3β-mediated Keap1/Nrf2/HO-1 signalling pathway. Front Genet. (2022) 13:865073. doi: 10.3389/fgene.2022.865073 35350242 PMC8957809

[92] Zheng Y Li L Chen H Zheng Y Tan X Zhang G. Luteolin exhibits synergistic therapeutic efficacy with erastin to induce ferroptosis in colon cancer cells through the HIC1-mediated inhibition of GPX4 expression. Free Radic Biol Med. (2023) 208:530–44. doi: 10.1016/j.freeradbiomed.2023.09.014 37717793

[93] Fu W Xu L Chen Y Zhang Z Chen S Li Q. Luteolin induces ferroptosis in prostate cancer cells by promoting TFEB nuclear translocation and increasing ferritinophagy. Prostate. (2024) 84:223–36. doi: 10.1002/pros.24642 37904332

[94] Wu Z Qu Q. Mechanism of luteolin induces ferroptosis in nasopharyngeal carcinoma cells. J Toxicol Sci. (2024) 49:399–408. doi: 10.2131/jts.49.399 39231684

[95] Han S Lin F Qi Y Liu C Zhou L Xia Y. HO-1 contributes to luteolin-triggered ferroptosis in clear cell renal cell carcinoma via increasing the labile iron pool and promoting lipid peroxidation. Oxid Med Cell Longev. (2022) 2022:3846217. doi: 10.1155/2022/3846217 35656025 PMC9153929

[96] Tang X Ding H Liang M Chen X Yan Y Wan N. Curcumin induces ferroptosis in non-small-cell lung cancer via activating autophagy. Thorac Cancer. (2021) 12:1219–30. doi: 10.1111/1759-7714.13904 PMC8046146 33656766

[97] Xu B Zhou L Zhang Q. Curcumin inhibits the progression of non-small cell lung cancer by regulating DMRT3/SLC7A11 axis. Mol Biotechnol. (2024) 67(5):1880–92. doi: 10.1007/s12033-024-01166-x 38744789

[98] Wu L Xu G Li N Zhu L Shao G. Curcumin Analog, HO-3867, Induces Both Apoptosis and Ferroptosis via Multiple Mechanisms in NSCLC Cells with Wild-Type p53. Evid Based Complement Alternat Med. (2023) 2023:8378581. doi: 10.1155/2023/8378581 36814470 PMC9940973

[99] Zhou J Zhang L Yan J Hou A Sui W Sun M. Curcumin induces ferroptosis in A549 CD133+ Cells through the GSH-GPX4 and FSP1-CoQ10-NAPH pathways. Discov Med. (2023) 35:251–63. doi: 10.24976/Discov.Med.202335176.26 37272092

[100] Yuan C Fan R Zhu K Wang Y Xie W Liang Y. Curcumin induces ferroptosis and apoptosis in osteosarcoma cells by regulating Nrf2/GPX4 signaling pathway. Exp Biol Med (Maywood). (2023) 248:2183–97. doi: 10.1177/15353702231220670 PMC10903231 38166505

[101] Lin H Chen X Zhang C Yang T Deng Z Song Y. EF24 induces ferroptosis in osteosarcoma cells through HMOX1. BioMed Pharmacother. (2021) 136:111202. doi: 10.1016/j.biopha.2020.111202 33453607

[102] Chen M Tan AH Li J. Curcumin represses colorectal cancer cell proliferation by triggering ferroptosis *via* PI3K/Akt/mTOR signaling. Nutr Cancer. (2023) 75:726–33. doi: 10.1080/01635581.2022.2139398 36346025

[103] Miyazaki K Xu C Shimada M Goel A. Curcumin and andrographis exhibit anti-tumor effects in colorectal cancer via activation of ferroptosis and dual suppression of glutathione peroxidase-4 and ferroptosis suppressor protein-1. Pharm (Basel). (2023) 16:383. doi: 10.3390/ph16030383 PMC10055708 36986483

[104] Xin W Zhang Y. Curcumin activates the JNK signaling pathway to promote ferroptosis in colon cancer cells. Chem Biol Drug Des. (2024) 103:e14468. doi: 10.1111/cbdd.14468 38443754

[105] Ming T Lei J Peng Y Wang M Liang Y Tang S. Curcumin suppresses colorectal cancer by induction of ferroptosis via regulation of p53 and solute carrier family 7 member 11/glutathione/glutathione peroxidase 4 signaling axis. Phytother Res. (2024) 38:3954–72. doi: 10.1002/ptr.8258 38837315

[106] Zheng X Liu J Hu W Jiang B Zhou X Zhang M. Curcumin induces autophagy-mediated ferroptosis by targeting the PI3K/AKT/mTOR signaling pathway in gastric cancer. Turk J Gastroenterol. (2024) 35:625–33. doi: 10.5152/tjg.2024.23526 PMC11363205 39150386

[107] Cao X Li Y Wang Y Yu T Zhu C Zhang X. Curcumin suppresses tumorigenesis by ferroptosis in breast cancer. PloS One. (2022) 17:e0261370. doi: 10.1371/journal.pone.0261370 35041678 PMC8765616

[108] Li R Zhang J Zhou Y Gao Q Wang R Fu Y. Transcriptome investigation and *in vitro* verification of curcumin-induced HO-1 as a feature of ferroptosis in breast cancer cells. Oxid Med Cell Longev. (2020) 2020:3469840. doi: 10.1155/2020/3469840 33294119 PMC7691002

[109] Consoli V Sorrenti V Pittalà V Greish K D’Amico AG Romeo G. Heme oxygenase modulation drives ferroptosis in TNBC cells. Int J Mol Sci. (2022) 23:5709. doi: 10.3390/ijms23105709 35628518 PMC9143660

[110] Chen H Li Z Xu J Zhang N Chen J Wang G. Curcumin induces ferroptosis in follicular thyroid cancer by upregulating HO-1 expression. Oxid Med Cell Longev. (2023) 2023:6896790. doi: 10.1155/2023/6896790 36691638 PMC9867595

[111] Xu B Zhu WJ Peng YJ Cheng SD. Curcumin reverses the sunitinib resistance in clear cell renal cell carcinoma (ccRCC) through the induction of ferroptosis via the *ADAMTS18* gene. Transl Cancer Res. (2021) 10:3158–67. doi: 10.21037/tcr-21-227 PMC8797884 35116623

[112] Shi M Zhang MJ Yu Y Ou R Wang Y Li H. Curcumin derivative NL01 induces ferroptosis in ovarian cancer cells via HCAR1/MCT1 signaling. Cell Signal. (2023) 109:110791. doi: 10.1016/j.cellsig.2023.110791 37406786

[113] Hurst WJ Glinski JA Miller KB Apgar J Davey MH Stuart DA. Survey of the trans-resveratrol and trans-piceid content of cocoa-containing and chocolate products. J Agric Food Chem. (2008) 56:8374–8. doi: 10.1021/jf801297w 18759443

[114] Zhang Z Ji Y Hu N Yu Q Zhang X Li J. Ferroptosis-induced anticancer effect of resveratrol with a biomimetic nano-delivery system in colorectal cancer treatment. Asian J Pharm Sci. (2022) 17:751–66. doi: 10.1016/j.ajps.2022.07.006 PMC9640689 36382309

[115] Jia M Tan X Yuan Z Zhu W Yan P. Nanoliposomes encapsulated rapamycin/resveratrol to induce apoptosis and ferroptosis for enhanced colorectal cancer therapy. J Pharm Sci. (2024) 113:2565–74. doi: 10.1016/j.xphs.2024.05.015 38768753

[116] Shan G Minchao K Jizhao W Rui Z Guangjian Z Jin Z. Resveratrol improves the cytotoxic effect of CD8^+^ T cells in the tumor microenvironment by regulating HMMR/Ferroptosis in lung squamous cell carcinoma. J Pharm BioMed Anal. (2023) 229:115346. doi: 10.1016/j.jpba.2023.115346 37001272

[117] Wang S Wang R Hu D Zhang C Cao P Huang J. Epigallocatechin gallate modulates ferroptosis through downregulation of tsRNA-13502 in non-small cell lung cancer. Cancer Cell Int. (2024) 24:200. doi: 10.1186/s12935-024-03391-5 38840243 PMC11155022

[118] Li F Hao S Gao J Jiang P. EGCG alleviates obesity-exacerbated lung cancer progression by STAT1/SLC7A11 pathway and gut microbiota. J Nutr Biochem. (2023) 120:109416. doi: 10.1016/j.jnutbio.2023.109416 37451475

[119] Liu CM An L Wu Z Ouyang AJ Su M Shao Z. 6-Gingerol suppresses cell viability, migration and invasion via inhibiting EMT, and inducing autophagy and ferroptosis in LPS-stimulated and LPS-unstimulated prostate cancer cells. Oncol Lett. (2022) 23:187. doi: 10.3892/ol.2022.13307 35527779 PMC9073581

[120] Tsai Y Xia C Sun Z. The Inhibitory Effect of 6-Gingerol on Ubiquitin-Specific Peptidase 14 Enhances Autophagy-Dependent Ferroptosis and Anti-Tumor *in vivo* and *in vitro* . Front Pharmacol. (2020) 11:598555. doi: 10.3389/fphar.2020.598555 33281606 PMC7691590

[121] Ma RH Ni ZJ Thakur K Cespedes-Acuña CL Zhang JG Wei ZJ. Transcriptome and proteomics conjoint analysis reveal metastasis inhibitory effect of 6-shogaol as ferroptosis activator through the PI3K/AKT pathway in human endometrial carcinoma *in vitro* and in *vivo* . Food Chem Toxicol. (2022) 170:113499. doi: 10.1016/j.fct.2022.113499 36341865

[122] Du J Krishnamoorthy K Ramabhai V Yang D. Targeting ferroptosis as a therapeutic implication in lung cancer treatment by a novel naphthoquinone inducer: juglone. Mol Biotechnol. (2024) 66:1071–81. doi: 10.1007/s12033-023-01004-6 38057629

[123] Karki N Aggarwal S Laine RA Greenway F Losso JN. Cytotoxicity of juglone and thymoquinone against pancreatic cancer cells. Chem Biol Interact. (2020) 327:109142. doi: 10.1016/j.cbi.2020.109142 32610056 PMC9115841

[124] Zhang YY Ni ZJ Elam E Zhang F Thakur K Wang S. Juglone, a novel activator of ferroptosis, induces cell death in endometrial carcinoma Ishikawa cells. Food Funct. (2021) 12:4947–59. doi: 10.1039/d1fo00790d 34100505

[125] Wang ZQ Li YQ Wang DY Shen YQ. Natural product piperlongumine inhibits proliferation of oral squamous carcinoma cells by inducing ferroptosis and inhibiting intracellular antioxidant capacity. Transl Cancer Res. (2023) 12:2911–22. doi: 10.21037/tcr-22-1494 PMC10643964 37969394

[126] Yang Y Sun S Xu W Zhang Y Yang R Ma K. Piperlongumine inhibits thioredoxin reductase 1 by targeting selenocysteine residues and sensitizes cancer cells to erastin. Antioxidants (Basel). (2022) 11:710. doi: 10.3390/antiox11040710 35453395 PMC9030593

[127] Yamaguchi Y Kasukabe T Kumakura S. Piperlongumine rapidly induces the death of human pancreatic cancer cells mainly through the induction of ferroptosis. Int J Oncol. (2018) 52:1011–22. doi: 10.3892/ijo.2018.4259 29393418

[128] Roh JL Kim EH Jang H Shin D. Nrf2 inhibition reverses the resistance of cisplatin-resistant head and neck cancer cells to artesunate-induced ferroptosis. Redox Biol. (2017) 11:254–62. doi: 10.1016/j.redox.2016.12.010 PMC5198738 28012440

[129] Shin D Kim EH Lee J Roh JL. Nrf2 inhibition reverses resistance to GPX4 inhibitor-induced ferroptosis in head and neck cancer. Free Radic Biol Med. (2018) 129:454–62. doi: 10.1016/j.freeradbiomed.2018.10.426 30339884

[130] Sun X Ou Z Chen R Niu X Chen D Kang R. Activation of the p62-Keap1-NRF2 pathway protects against ferroptosis in hepatocellular carcinoma cells. Hepatology. (2016) 63:173–84. doi: 10.1002/hep.28251 PMC4688087 26403645

[131] Liu XY Wei DG Li RS. Capsaicin induces ferroptosis of NSCLC by regulating SLC7A11/GPX4 signaling *in vitro* . Sci Rep. (2022) 12:11996. doi: 10.1038/s41598-022-16372-3 35835852 PMC9283462

[132] Hacioglu C Kar F. Capsaicin induces redox imbalance and ferroptosis through ACSL4/GPx4 signaling pathways in U87-MG and U251 glioblastoma cells. Metab Brain Dis. (2023) 38:393–408. doi: 10.1007/s11011-022-00983-w 35438378

[133] Cong Z Zhao Q Yang B Cong D Zhou Y Lei X. Ginsenoside rh3 inhibits proliferation and induces apoptosis of colorectal cancer cells. Pharmacology. (2020) 105:329–38. doi: 10.1159/000503821 31671429

[134] Yang T Xu W Wei X Zhang Z Sun Y Liu H. Determination of ginsenoside Rh3 in rat plasma by LC-MS/MS and its application to a pharmacokinetic study. BioMed Chromatogr. (2022) 36:e5268. doi: 10.1002/bmc.v36.2 34676576

[135] Baek NI Kim DS Lee YH Park JD Lee CB Kim SI. Ginsenoside Rh4, a genuine dammarane glycoside from Korean red ginseng. Planta Med. (1996) 62:86–7. doi: 10.1055/s-2006-957816 8720394

[136] Deng X Zhao J Qu L Duan Z Fu R Zhu C. Ginsenoside Rh4 suppresses aerobic glycolysis and the expression of PD-L1 via targeting AKT in esophageal cancer. Biochem Pharmacol. (2020) 178:114038. doi: 10.1016/j.bcp.2020.114038 32422139

[137] Wu Q Deng J Fan D Duan Z Zhu C Fu R. Ginsenoside Rh4 induces apoptosis and autophagic cell death through activation of the ROS/JNK/p53 pathway in colorectal cancer cells. Biochem Pharmacol. (2018) 148:64–74. doi: 10.1016/j.bcp.2017.12.004 29225132

[138] Gao XF Zhang JJ Gong XJ Li KK Zhang LX Li W. Ginsenoside Rg5: A review of anticancer and neuroprotection with network pharmacology approach. Am J Chin Med. (2022) 50:2033–56. doi: 10.1142/S0192415X22500872 36222119

[139] Oh JM Lee J Im WT Chun S. Ginsenoside rk1 induces apoptosis in neuroblastoma cells through loss of mitochondrial membrane potential and activation of caspases. Int J Mol Sci. (2019) 20:1213. doi: 10.3390/ijms20051213 30862004 PMC6429382

[140] Gao S Fang C Wang T Lu W Wang N Sun L. The effect of ginsenoside Rg3 combined with chemotherapy on immune function in non-small cell lung cancer: A systematic review and meta-analysis of randomized controlled trials. Med (Baltimore). (2023) 102:e33463. doi: 10.1097/MD.0000000000033463 PMC10082263 37026927

[141] Majewska-Wierzbicka M Czeczot H. Przeciwnowotworowe działanie flawonoidów [Anticancer activity of flavonoids]. Pol Merkur Lekarski. (2012) 33:364–9. 23437710

[142] Islam A Islam MS Rahman MK Uddin MN Akanda MR. The pharmacological and biological roles of eriodictyol. Arch Pharm Res. (2020) 43:582–92. doi: 10.1007/s12272-020-01243-0 32594426

[143] Mehranfard N Ghasemi M Rajabian A Ansari L. Protective potential of naringenin and its nanoformulations in redox mechanisms of injury and disease. Heliyon. (2023) 9:e22820. doi: 10.1016/j.heliyon.2023.e22820 38058425 PMC10696200

[144] Harmon AW Patel YM. Naringenin inhibits glucose uptake in MCF-7 breast cancer cells: a mechanism for impaired cellular proliferation. Breast Cancer Res Treat. (2004) 85:103–10. doi: 10.1023/B:BREA.0000025397.56192.e2 15111768

[145] Lian GY Wang QM Tang PM Zhou S Huang XR Lan HY. Combination of asiatic acid and naringenin modulates NK cell anti-cancer immunity by rebalancing Smad3/Smad7 Signaling. Mol Ther. (2018) 26:2255–66. doi: 10.1016/j.ymthe.2018.06.016 PMC6127879 30017880

[146] Zhu J Sun R Yan C Sun K Gao L Zheng B. Hesperidin mitigates oxidative stress-induced ferroptosis in nucleus pulposus cells via Nrf2/NF-κB axis to protect intervertebral disc from degeneration. Cell Cycle. (2023) 22:1196–214. doi: 10.1080/15384101.2023.2200291 PMC10193898 37055945

[147] Sohel M Sultana H Sultana T Al Amin M Aktar S Ali MC. Chemotherapeutic potential of hesperetin for cancer treatment, with mechanistic insights: A comprehensive review. Heliyon. (2022) 8:e08815. doi: 10.1016/j.heliyon.2022.e08815 35128104 PMC8810372

[148] Imran M Aslam Gondal T Atif M Shahbaz M Batool Qaisarani T Hanif Mughal M. Apigenin as an anticancer agent. Phytother Res. (2020) 34:1812–28. doi: 10.1002/ptr.6647 32059077

[149] Yao Z Gu Y Zhang Q Liu L Meng G Wu H. Estimated daily quercetin intake and association with the prevalence of type 2 diabetes mellitus in Chinese adults. Eur J Nutr. (2019) 58:819–30. doi: 10.1007/s00394-018-1713-2 29754250

[150] Huang J Chen J Li J. Quercetin promotes ATG5-mediating autophagy-dependent ferroptosis in gastric cancer. J Mol Histol. (2024) 55:211–25. doi: 10.1007/s10735-024-10186-5 38441713

[151] Zhu YW Liu CL Li XM Shang Y. Quercetin induces ferroptosis by inactivating mTOR/S6KP70 pathway in oral squamous cell carcinoma. Toxicol Mech Methods. (2024) 34:669–75. doi: 10.1080/15376516.2024.2325989 38736312

[152] Lin ZH Liu Y Xue NJ Zheng R Yan YQ Wang ZX. Quercetin protects against MPP+/MPTP-induced dopaminergic neuron death in parkinson’s disease by inhibiting ferroptosis. Oxid Med Cell Longev. (2022) 2022:7769355. doi: 10.1155/2022/7769355 36105483 PMC9467739

[153] Nguyen-Ngo C Salomon C Quak S Lai A Willcox JC Lappas M. Nobiletin exerts anti-diabetic and anti-inflammatory effects in an in vitro human model and in vivo murine model of gestational diabetes. Clin Sci (Lond). (2020) 134:571–92. doi: 10.1042/CS20191099 32129440

[154] Chen Z Kong S Song F Li L Jiang H. Pharmacokinetic study of luteolin, apigenin, chrysoeriol and diosmetin after oral administration of Flos Chrysanthemi extract in rats. Fitoterapia. (2012) 83:1616–22. doi: 10.1016/j.fitote.2012.09.011 PMC7127355 22999990

[155] Lim SH Jung SK Byun S Lee EJ Hwang JA Seo SG. Luteolin suppresses UVB-induced photoageing by targeting JNK1 and p90 RSK2. J Cell Mol Med. (2013) 17:672–80. doi: 10.1111/jcmm.12050 PMC3822820 23551430

[156] Kim Y Clifton P. Curcumin, cardiometabolic health and dementia. Int J Environ Res Public Health. (2018) 15:2093. doi: 10.3390/ijerph15102093 30250013 PMC6210685

[157] Kotha RR Luthria DL. Curcumin: biological, pharmaceutical, nutraceutical, and analytical aspects. Molecules. (2019) 24:2930. doi: 10.3390/molecules24162930 31412624 PMC6720683

[158] Howells LM Iwuji COO Irving GRB Barber S Walter H Sidat Z. Curcumin combined with FOLFOX chemotherapy is safe and tolerable in patients with metastatic colorectal cancer in a randomized phase IIa trial. J Nutr. (2019) 149:1133–9. doi: 10.1093/jn/nxz029 PMC6602900 31132111

[159] Pilankar A Singhavi H Raghuram GV Siddiqui S Khare NK Jadhav V. A pro-oxidant combination of resveratrol and copper down-regulates hallmarks of cancer and immune checkpoints in patients with advanced oral cancer: Results of an exploratory study (RESCU 004). Front Oncol. (2022) 12:1000957. doi: 10.3389/fonc.2022.1000957 36185249 PMC9525028

[160] Li G Zhou C Wang L Zheng Y Zhou B Li G. MitoCur-1 induces ferroptosis to reverse vemurafenib resistance in melanoma through inhibition of USP14. Pigment Cell Melanoma Res. (2024) 37:316–28. doi: 10.1111/pcmr.13150 37985430

[161] Burns J Yokota T Ashihara H Lean ME Crozier A. Plant foods and herbal sources of resveratrol. J Agric Food Chem. (2002) 50:3337–40. doi: 10.1021/jf0112973 12010007

[162] Liu J Gao W Sheng Y Sun J Wen D. Resveratrol drives ferroptosis of acute myeloid leukemia cells through Hsa-miR-335-5p/NFS1/GPX4 pathway in a ROS-dependent manner. Cell Mol Biol (Noisy-le-grand). (2023) 69:131–7. doi: 10.14715/cmb/2023.69.7.21 37715395

[163] Zhu T Jin S Tong D Liu X Liu Y Zheng J. Enhancing the anti-tumor efficacy of NK cells on canine mammary tumors through resveratrol activation. Anim (Basel). (2024) 14:1636. doi: 10.3390/ani14111636 PMC11171074 38891683

[164] Chakrawarti L Agrawal R Dang S Gupta S Gabrani R. Therapeutic effects of EGCG: a patent review. Expert Opin Ther Pat. (2016) 26:907–16. doi: 10.1080/13543776.2016.1203419 27338088

[165] Konmun J Danwilai K Ngamphaiboon N Sripanidkulchai B Sookprasert A Subongkot S. A phase II randomized double-blind placebo-controlled study of 6-gingerol as an anti-emetic in solid tumor patients receiving moderately to highly emetogenic chemotherapy. Med Oncol. (2017) 34:69. doi: 10.1007/s12032-017-0931-4 28349496

[166] Ahmad T Suzuki YJ. Juglone in oxidative stress and cell signaling. Antioxidants (Basel). (2019) 8:91. doi: 10.3390/antiox8040091 30959841 PMC6523217

[167] Li B Zu M Jiang A Cao Y Wu J Shahbazi MA. Magnetic natural lipid nanoparticles for oral treatment of colorectal cancer through potentiated antitumor immunity and microbiota metabolite regulation. Biomaterials. (2024) 307:122530. doi: 10.1016/j.biomaterials.2024.122530 38493672

[168] Heinrich M Mah J Amirkia V. Alkaloids used as medicines: structural phytochemistry meets biodiversity-an update and forward look. Molecules. (2021) 26:1836. doi: 10.3390/molecules26071836 33805869 PMC8036335

[169] Debnath B Singh WS Das M Goswami S Singh MK Maiti D. Role of plant alkaloids on human health: A review of biological activities. Materials Today Chem. (2018) 9:56–72. CA1. doi: 10.1016/j.mtchem.2018.05.001

[170] Piska K Gunia-Krzyżak A Koczurkiewicz P Wójcik-Pszczoła K Pękala E. Piperlongumine (piplartine) as a lead compound for anticancer agents - Synthesis and properties of analogues: A mini-review. Eur J Med Chem. (2018) 156:13–20. doi: 10.1016/j.ejmech.2018.06.057 30006159

[171] Raj L Ide T Gurkar AU Foley M Schenone M Li X. Selective killing of cancer cells by a small molecule targeting the stress response to ROS. Nature. (2011) 475:231–4. doi: 10.1038/nature15370 PMC3316487 21753854

[172] Mohamadi N Sharififar F Pournamdari M Ansari M. A review on biosynthesis, analytical techniques, and pharmacological activities of trigonelline as a plant alkaloid. J Diet Suppl. (2018) 15:207–22. doi: 10.1080/19390211.2017.1329244 28816550

[173] European Commission Health & Consumer Protection Directorate-General. Opinion of the Scientific Committee on Food on Capsaicin. Brussel, Belgium: European Commission Health & Consumer Protection Directorate-General (2002) p. 1–12. Google Scholar.

[174] Deng X Gui Y Zhao L Li N Li L. Arvanil induces ferroptosis of hepatocellular carcinoma by binding to MICU1. Cancer Gene Ther. (2024) 31:148–57. doi: 10.1038/s41417-023-00690-3 37985721

[175] Boden A Lusque A Lodin S Bourgouin M Mauries V Moreau C. Study protocol of the TEC-ORL clinical trial: a randomized comparative phase II trial investigating the analgesic activity of capsaicin vs Laroxyl in head and neck Cancer survivors presenting with neuropathic pain sequelae. BMC Cancer. (2022) 22:1260. doi: 10.1186/s12885-022-10348-2 36471253 PMC9720988

[176] Liu R Rong G Liu Y Huang W He D Lu R. Delivery of apigenin-loaded magnetic Fe2O3/Fe3O4@mSiO2 nanocomposites to A549 cells and their antitumor mechanism. Mater Sci Eng C Mater Biol Appl. (2021) 120:111719. doi: 10.1016/j.msec.2020.111719 33545870

[177] Chen R Jiang Z Cheng Y Ye J Li S Xu Y. Multifunctional iron-apigenin nanocomplex conducting photothermal therapy and triggering augmented immune response for triple negative breast cancer. Int J Pharm. (2024) 655:124016. doi: 10.1016/j.ijpharm.2024.124016 38503397

[178] Mu M Wang Y Zhao S Li X Fan R Mei L. Engineering a pH/glutathione-responsive tea polyphenol nanodevice as an apoptosis/ferroptosis-inducing agent. ACS Appl Bio Mater. (2020) 3:4128–38. doi: 10.1021/acsabm.0c00225 35025415

[179] Xu R Yang J Qian Y Deng H Wang Z Ma S. Ferroptosis/pyroptosis dual-inductive combinational anti-cancer therapy achieved by transferrin decorated nanoMOF. Nanoscale Horiz. (2021) 6:348–56. doi: 10.1039/d0nh00674b 33687417

[180] Shu L Luo P Chen Q Liu J Huang Y Wu C. Fibroin nanodisruptor with Ferroptosis-Autophagy synergism is potent for lung cancer treatment. Int J Pharm. (2024) 664:124582. doi: 10.1016/j.ijpharm.2024.124582 39142466

[181] Chen F Kang R Tang D Liu J. Ferroptosis: principles and significance in health and disease. J Hematol Oncol. (2024) 17:41. doi: 10.1186/s13045-024-01564-3 38844964 PMC11157757

[182] Li S Jiang Q Liu S Zhang Y Tian Y Song C. A DNA nanorobot functions as a cancer therapeutic in response to a molecular trigger in *vivo* . Nat Biotechnol. (2018) 36:258–64. doi: 10.1038/nbt.4071 29431737

[183] Shoba G Joy D Joseph T Majeed M Rajendran R Srinivas PS. Influence of piperine on the pharmacokinetics of curcumin in animals and human volunteers. Planta Med. (1998) 64:353–6. doi: 10.1055/s-2006-957450 9619120

[184] Zhou Y Zhang T Wang X Wei X Chen Y Guo L. Curcumin modulates macrophage polarization through the inhibition of the toll-like receptor 4 expression and its signaling pathways. Cell Physiol Biochem. (2015) 36:631–41. doi: 10.1159/000430126 25998190

[185] Gonzales AM Orlando RA. Curcumin and resveratrol inhibit nuclear factor-kappaB-mediated cytokine expression in adipocytes. Nutr Metab (Lond). (2008) 5:17. doi: 10.1186/1743-7075-5-17 18549505 PMC2441623

[186] Hassaninasab A Hashimoto Y Tomita-Yokotani K Kobayashi M. Discovery of the curcumin metabolic pathway involving a unique enzyme in an intestinal microorganism. Proc Natl Acad Sci U S A. (2011) 108:6615–20. doi: 10.1073/pnas.1016217108 PMC3080977 21467222

[187] Keyvani-Ghamsari S Khorsandi K Gul A. Curcumin effect on cancer cells’ multidrug resistance: An update. Phytother Res. (2020) 34:2534–56. doi: 10.1002/ptr.6703 32307747

[188] Hussain Y Islam L Khan H Filosa R Aschner M Javed S. Curcumin-cisplatin chemotherapy: A novel strategy in promoting chemotherapy efficacy and reducing side effects. Phytother Res. (2021) 35:6514–29. doi: 10.1002/ptr.7225 34347326

[189] Wang W Li M Wang L Chen L Goh BC. Curcumin in cancer therapy: Exploring molecular mechanisms and overcoming clinical challenges. Cancer Lett. (2023) 570:216332. doi: 10.1016/j.canlet.2023.216332 37541540

