# Correction: Food-derived compounds targeting ferroptosis for cancer therapy: from effects to mechanisms

**DOI:** 10.3389/fonc.2025.1644727

**Published:** 2025-07-17

**Authors:** Jin-Wei Zhao, Wei-Yi Zhao, Zhongyang Yu

**Affiliations:** ^1^ Department of Hepatopancreatobiliary Surgery of Second Hospital of Jilin University, Jilin University, Changchun, China; ^2^ Department of Cardiology, Jilin Airport Hospital, Jilin, China; ^3^ State Key Laboratory of Cardiovascular Diseases, Shanghai East Hospital, School of Medicine, Tongji University, Shanghai, China

**Keywords:** food-derived bioactive ingredients, ferroptosis inducer, synergistic mechanism, cancer, treatment

Affiliation “State Key Laboratory of Cardiovascular Diseases, Shanghai East Hospital, School of Medicine, Tongji University, Shanghai, China” was omitted for author Zhongyang Yu. This affiliation has now been added for author Zhongyang Yu.

The original version of this article has been updated.

